# Low-level features predict perceived similarity for naturalistic images

**DOI:** 10.1167/jov.25.12.11

**Published:** 2025-10-07

**Authors:** Emily J. A-Izzeddin, Thomas S. A. Wallis, Jason B. Mattingley, William J. Harrison

**Affiliations:** 1 Department of Experimental Psychology, Justus Liebig University Giessen, Giessen, Germany; 2 Queensland Brain Institute, University of Queensland, St Lucia, Queensland, Australia; 3 Center for Mind, Brain and Behavior (CMBB), Universities of Marburg, Giessen, and Darmstadt, Marburg, Germany; 4 Institute of Psychology & Centre for Cognitive Science, Technical University of Darmstadt, Darmstadt, Germany; 5 School of Psychology, University of Queensland, St Lucia, Queensland, Australia; 6 School of Health, University of the Sunshine Coast, Sippy Downs, Queensland, Australia

**Keywords:** naturalistic images, scene perception, computational modelling, image statistics

## Abstract

The mechanisms by which humans perceptually organize individual regions of a visual scene to generate a coherent scene representation remain largely unknown. Our perception of statistical regularities has been relatively well-studied in simple stimuli, and explicit computational mechanisms that use low-level image features (e.g., luminance, contrast energy) to explain these perceptions have been described. Here, we investigate to what extent observers can effectively use such low-level information present in isolated naturalistic scene regions to facilitate associations between said regions. Across two experiments, participants were shown an isolated reference patch, then required to select which of two subsequently presented patches came from the same scene as the reference (two-alternative forced choice method). In Experiment 1, participants made their judgments based on unaltered image patches, and were consistently above chance when performing such association judgments. Additionally, participants’ responses were well-predicted by a generalized linear multilevel model using predictors based on low-level feature similarity metrics (specifically, pixel-wise luminance and phase-invariant structure correlations). In Experiment 2, participants were presented with unaltered image regions, thresholded image regions, or regions reduced to only their edge content. Performance for thresholded and edge regions was significantly poorer than for unaltered image regions. Nonetheless, the model still correlated well with participants’ judgments. Our findings suggest that image region associations can be accounted for using low-level feature correlations, suggesting such basic features are strongly associated with those underlying judgments made for complex visual stimuli.

## Introduction

Fundamental to our daily functioning is our ability to interpret the complexity of our visual surroundings, which in turn guides how we behave. When forming such interpretations, the majority of information is typically yielded from the current visual scene. Visual information is critical for efficient deduction of many task-relevant factors (Frazor & Geisler, 2006; Torralba & Oliva, 2003), such as determining if we have turned onto the right street on our way to work or finding the right product in a supermarket aisle. Critical to performing such efficient visual interpretations, on top of broader contextual information across the entire visual field, we process our environment via eye movements. Eye movements shift the fovea to provide high-definition input for various areas of the scene (Akbas & Eckstein, 2017; Mirza, Adams, Mathys, & Friston, 2016; Noton & Stark, 1971). Based on this information gathering, the visual system must construct a coherent representation of each scene by somehow integrating multiple relevant regions of visual space—a computational challenge that has been thoroughly discussed but not yet solved (Berens, Joensen, & Horner, 2021; Chen, Cichy, & Kaiser, 2023; Cohen et al., 1999; Goh et al., 2004; Harrison, Stead, Wallis, Bex, & Mattingley, 2024; Hassabis & Maguire, 2007; Robertson, Hermann, Mynick, Kravitz, & Kanwisher, 2016; Singer & Gray, 1995; Steel, Billings, Silson, & Robertson, 2021; Treisman, 1998). However, it remains largely unclear how we perform this complex scene mapping process or, in the simplest case, how we associate two different regions of visual space with one another. Hence, in the current study, we investigated observers’ scene region association judgments on two isolated regions. Specifically, we were interested in how available information in isolated scene regions facilitates such associations, giving insight into whether and how such information may contribute to broader scene mapping processes.

Being able to perform scene region associations can have considerable implications for informing our current behaviour and goals. By scene region associations, we refer to observers’ ability to determine whether distinct subregions belong to the same scene. In performing scene–region associations, we also inherently dissociate those regions from adjacent scenes or environments. Imagine standing in a central location of a house; you might be able to see, simultaneously, the living room, the dining room, the kitchen, and perhaps even other rooms through adjoining doors. This in and of itself can be considered to be one coherent scene (i.e., the inside of a house). However, if you were asked to find a sweater in the living room, it becomes functionally beneficial for you to focus on the smaller living room scene within the broader house scene. Therefore, to guide basic functions such as visual search, you need to delineate the boundary of the living room from other visible rooms and, critically, to associate the relevant regions of the living room with each other. Here, we emphasize the importance of integrating scene regions, as opposed to segmenting a scene. Such a process of refining the scope of the scene currently being considered demonstrates the flexible and task/goal-oriented process of scene defining (Henderson & Hollingworth, 1999). However, the visual processing invoked to facilitate such scene-defining behaviour remains unclear.

There is an abundance of visual information available to us in naturalistic scenes, any subset of which we might conceivably use to associate regions of space (Henderson & Hollingworth, 1999). Heavy emphasis has been placed on the contribution of semantic visual information to scene processing, with consistent evidence for such high-level information being influential in visual object search and recognition (Bar, 2004; Bar & Ullman, 1993; Biederman, Mezzanotte, & Rabinowitz, 1982; Brockmole, Castelhano, & Henderson, 2006; Brockmole & Henderson, 2006a; Brockmole & Henderson, 2006b; Davenport & Potter, 2004; Eckstein, Drescher, & Shimozaki, 2006; Friedman, 1979; Henderson, 2003; Henderson, Weeks, Jr., & Hollingworth, 1999; Hidalgo-Sotelo, Oliva, & Torralba, 2005; Hollingworth & Henderson, 2000; Loftus & Mackworth, 1978; Neider & Zelinsky, 2006; Palmer, 1975). However, given that the semantic understanding of a scene follows from visual information, it is possible that semantics are, in some cases, redundant in scene processing. Instead, it is possible that lower-level visual information contributes uniquely to scene processing. Hence, beyond semantic visual information, there remains scope to further our understanding of scene processing by investigating the contribution of basic visual features.

**Figure 1.**
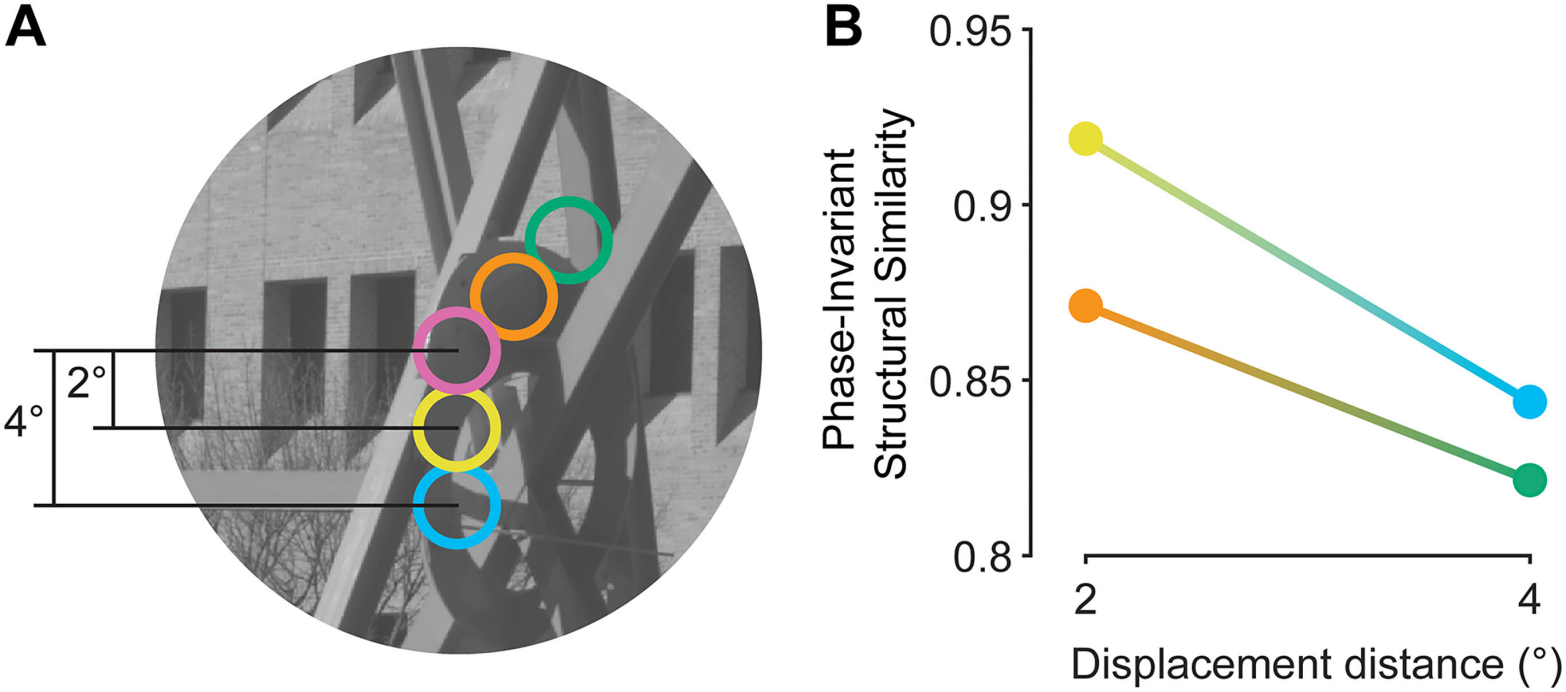
The impact of separation on image region correlations. (**A**) An illustration of different spatial offsets one can consider between image regions. For example, we can vary separation distance relative to the central pink region, comparing relatively spatially proximal (e.g., orange and yellow) vs. distant (e.g., blue and green) regions. Alternatively, we can vary separation azimuth, comparing cardinally (i.e., yellow and blue) vs. obliquely (i.e., orange and green) offset regions. (**B**) Similarity between the image regions in (**A**), as measured by “phase-invariant structural similarity” (Sebastian et al., 2017; see Section 3.7.2., GLMM, for more detail). Here, the actual phase-invariant structural similarity between the pink region and other regions is plotted on the *y* axis, with separation distance on the *x* axis, and separate lines for cardinal (yellow and blue) vs. oblique (orange and green) separations.

Low-level features are a clear candidate for information we might use to associate regions of space. Here, low-level features are conceptually defined as any image information that does not convey semantic meaning (Neri, 2014). Operationally, we define low-level features as any information that can be computed from simple oriented contrast filters. Low-level feature relationships between regions of space have been explored computationally (Field, 1987; Frazor & Geisler, 2006; Harrison, 2021; Simoncelli & Olshausen, 2001). Practically, such computations typically involve some form of image decomposition that measures particular image features, such as contrast energy, at different orientation/frequency bands. From this, image statistics for a specific image region can be calculated—for example, quantifying the prevalence of particular orientations. Resulting statistics can then be correlated with the statistics of a different image region to provide a measure of similarity between the two regions in question. Such similarity assessments have suggested that the strength of these relationships is largely dependent on the relative spatial position of the two regions being associated (Figure 1) (Field, 1987; Frazor & Geisler, 2006; Harrison, 2021; Simoncelli & Olshausen, 2001). For example, there is a clear influence of spatial separation, whereby there tend to be stronger relationships of image-computable metrics between spatially proximal regions of space than more distant regions on average (Figure 1B, displacement of 2° vs. 4°). Similarly, owing to the over-representation of cardinal orientations relative to obliques in nature, low-level feature relationships will be stronger with cardinal separation axes on average than oblique separation axes (Figure 1B, separate lines) (Coppola, Purves, McCoy, & Purves, 1998; Essock, DeFord, Hansen, & Sinai, 2003; Girshick, Landy, & Simoncelli, 2011; Hansen, Essock, Zheng, & Deford, 2003; Hansen & Essock, 2004; Harrison, 2021; Keil & Cristóbal, 2000). Hence, the computational capacity to associate regions of space based on low-level feature correlations is well-established.

Beyond computational investigations, there has been extensive research into our sensitivity to basic visual information. Key insights into visual sensitivity have emerged from work investigating perceptual priors (expectations rooted in the statistical regularities we observe in nature) for such basic visual information and their subsequent impact on perception (Series & Seitz, 2013; Summerfield & Egner, 2009). For example, we have a greater sensitivity to cardinal orientations as compared with obliques (Appelle, 1972; Berkley, Kitterle, & Watkins, 1975; Campbell, Kulikowski, & Levinson, 1966; Dakin, 2001; Dakin, Bex, Cass, & Watt, 2009; Dakin & Watt, 1997; de Gardelle, Kouider, & Sackur, 2010; Emsley, 1925; Girshick et al., 2011; Henderson & Hollingworth, 1999; Pratte, Park, Rademaker, & Tong, 2016; Westheimer & Beard, 1998) owing to a prior that reflects our greater exposure to cardinal orientations in nature as compared with obliques (Coppola et al., 1998; Essock et al., 2003; Girshick et al., 2011; Hansen et al., 2003; Hansen & Essock, 2004; Harrison, 2021; Keil & Cristóbal, 2000). However, although observers are clearly able to make perceptual judgments based on such low-level information, they have typically been probed with these visual features presented in isolation (e.g., oriented gratings or bars; Carandini, 2005; David, Vinje, & Gallant, 2004; Olshausen & Field, 2005; Sonkusare, Breakspear, & Guo, 2019). Therefore, documented judgments on basic visual features are not necessarily representative of our judgments on the complex visual information conveyed by a naturalistic scene. Hence, the question remains as to whether we are sensitive to low-level information to the extent that it can facilitate naturalistic image region associations.

Previous investigations have sought to understand the association between low-level feature distributions and processing in more naturalistic stimuli. For example, changes in low-level image statistics, such as edge density, phase, and contrast, have been shown to predict target detection in photographic images (Bex, 2010; Bex, Solomon, & Dakin, 2009; Rideaux et al., 2022; Sebastian, Abrams, & Geisler, 2017; Sebastian, Abrams, & Geisler, 2020; Spehar & Taylor, 2013; Wallis & Bex, 2012). Further, aesthetic preferences and complexity judgments on images seem to be aligned with stimuli that more closely reflect the typical underlying image statistics we experience in nature (Aks & Sprott, 1996; Knill, Field, & Kersten, 1990; Spehar, Clifford, Newell, & Taylor, 2003; Sprott, 1993; Taylor, Spehar, Hagerhall, & Van Donkelaar, 2011; Viengkham, Isherwood, & Spehar, 2022; Viengkham & Spehar, 2018). Such studies suggest that the low-level features present in naturalistic images are able to influence our perceptual experience.

**Figure 2.**
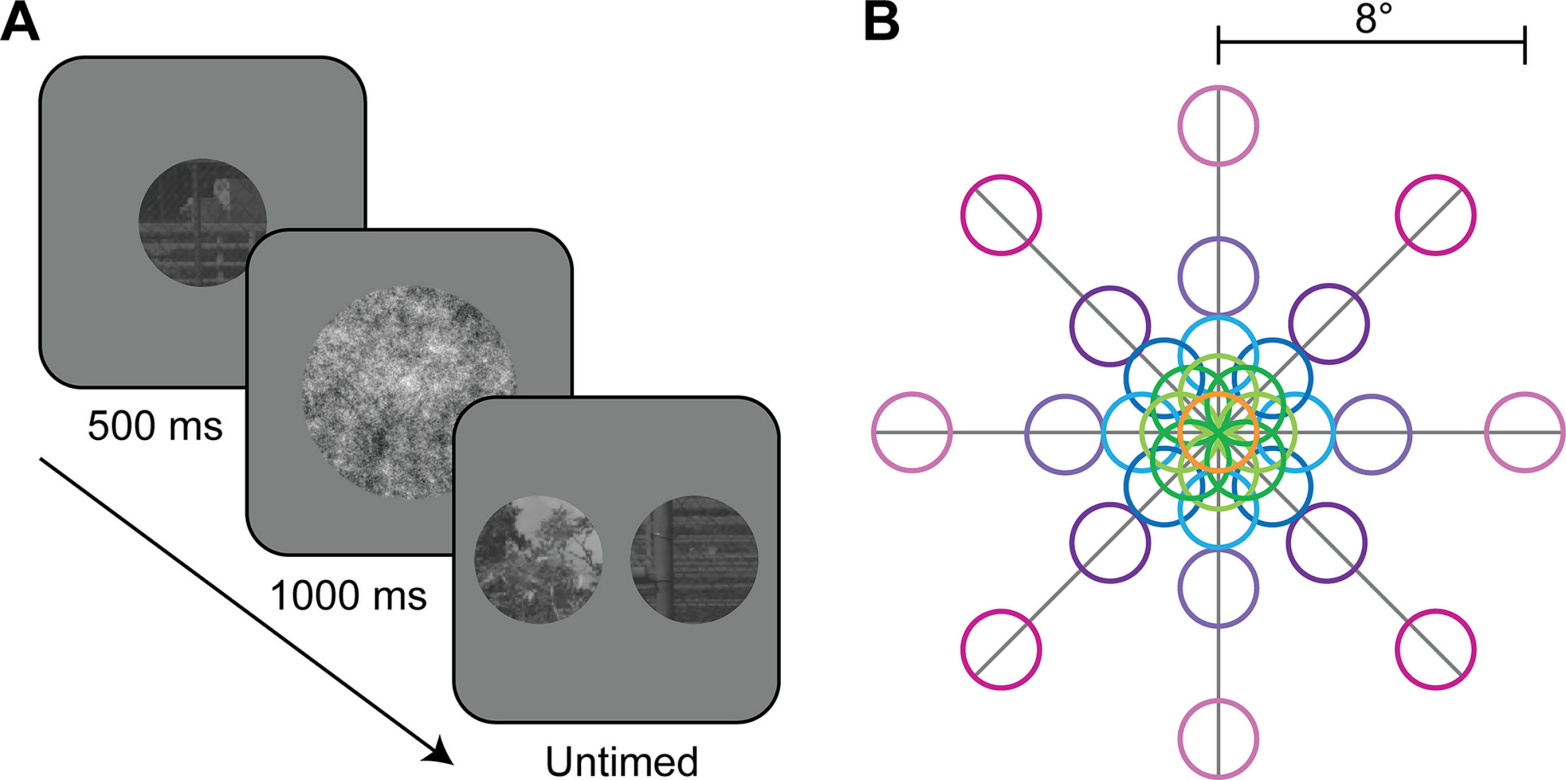
Experiment 1 paradigm overview. (**A**) Schematic of general trial structure. On each trial, participants viewed a central fixation point (not pictured) for 700 ms, followed by the reference patch for 500 ms, a random pink noise mask (not to scale) for 1,000 ms and, finally, the target (in this case, right) and foil (in this case, left) patches until a response was made. (**B**) To-scale schematic of possible target/foil spatial locations relative to the reference patch (orange, also the location of the 0° separation condition), manipulating separation distance (0°, 1°, 2°, 4°, and 8°) and azimuth (0°, 45°, 90°, 135°, 180°, 225°, 270°, and 315°).

In the present study, across two experiments, we investigated observers’ capacity to perform naturalistic scene region associations, and the predictability of their responses from low-level visual features. Participants viewed small image patches, windowed from broader naturalistic images, and indicated which two of three were drawn from the same broader scene (Figure 2A). By windowing the patches, we removed broader contextual information that could otherwise have been exploited to disambiguate the relationship between the patches. In this way, we encouraged participants to rely on the available low-level information (A-Izzeddin, Mattingley, & Harrison, 2024). Importantly, patches drawn from the same broader scene were selected based on various combinations of separation distances and azimuths, thereby altering their low-level feature relationships (Figure 2B; Field, 1987; Frazor & Geisler, 2006; Simoncelli & Olshausen, 2001) and allowing us to investigate the impact of spatial separations on image region association judgments. Crucially, we used computational modelling to predict observers’ capacity to perform naturalistic scene region associations based on low-level visual feature correlations. In doing so, we investigate whether reliance on more complex information is necessary for completing such scene region associations.

## Methods

### Participants

Two experiments were conducted with different participants in each experiment. We tested 40 participants in total (20 per experiment), aged 18-34. Participants were recruited through The University of Queensland's Psychology Research Participation Scheme, facilitated by Sona. All participants were naïve to the purpose of the experiments and reported normal or corrected-to-normal vision. Ethics approval was granted by The University of Queensland (Medicine), Low & Negligible Risk Ethics Sub-Committee, with all methods performed in accordance with the Declaration of Helsinki.

### General task design

A schematic for a typical trial is shown in Figure 2A. Participants were first shown a fixation point in the centre of the screen. The fixation point was followed by a “reference” patch, which was an image region cropped from a larger photograph. Following the reference patch, participants saw a random pink noise mask (to minimize any impact of retinal adaptation), followed by two new image patches—a “target” and a “foil”. The target patch was another image region cropped from the same larger photograph as the reference, and the foil was cropped from an entirely different photograph. Participants were asked to indicate which of the two (target vs. foil) came from the same larger photograph as the reference using the arrow keys on a standard computer keyboard. Participants did not receive feedback. Participants were only ever shown the isolated image regions, never the whole source images, thereby preventing insight into each scene's broad spatial arrangement. Therefore, participants had to rely on only the structures within the patches themselves.

### Stimuli

Stimuli were generated in the same manner across all experiments unless specified. Digital natural images were taken from a database of high-resolution 4256 × 2836-pixel color photos, cropped to 104 evenly-spaced constituent 1080 × 1080-pixel regions (henceforth “source images”; Burge & Geisler, 2011). For both experiments, each trial comprised of one reference, one target, and one foil stimulus. For each trial, two unique source images were selected—one as the source for the reference and target patches, and the other as the source for the foil. The 104 source images cropped from a single original image in Burge and Geisler's (2011) database contained overlapping content. Hence, the two source images on a given trial were selected to ensure they came from two different original images, preventing any overlap. Stimuli on each trial (i.e., the reference, target, and foil) were circular patches cropped from the selected 1080 × 1080 source images, subtending 2° of visual angle in diameter. All images were converted to greyscale, to ensure that only luminance-based image structure could be used to perform the task. It is likely that color information would provide a strong disambiguating cue, which was beyond the scope of the present work to examine. Images were converted to greyscale using MATLAB's rgb2gray() function, which transforms colored images into achromatic luminance images.

Reference patches were always cropped from the centre of the source image and were always presented to participants centrally. Original database images were taken of common scenes observed by the researchers at their university campus (Burge & Geisler, 2011). Hence, by cropping 1080 × 1080 regions of the original database photos and selecting one of these regions as the source image, any systematic ‘photographer’ bias which might bias the central region of the image (i.e., where the reference is drawn from) should have been eliminated. Target patches were cropped from one of 33 possible spatial locations relative to the reference patch (Figure 2B). To further account for any potential photographer bias, foil patches were drawn from their source images at matched spatial locations to the target patches on each trial. The 33 possible spatial locations for the target and foil patches were specified combinations of distance (1°, 2°, 4°, or 8° of visual angle) and azimuth (0°, 45°, 90°, 135°, 180°, 225°, 270°, or 315°) separations relative to the reference patch (i.e., the centre of the image). Additionally, a 0° separation distance condition was included, where the target patch was identical to the reference patch, to assess participants’ ability to perform the task when given identical region information. Target and foil patches were always presented simultaneously, separated either vertically or horizontally (counterbalanced across participants), with the side the target patch was on also balanced across trials.

### Apparatus

For both experiments, stimuli were generated on a Dell Precision T1700 computer (running Windows 7 Enterprise) with the Psychophysics Toolbox (3.0.17; Brainard, 1997; Pelli, 1997) for MATLAB (R2020b). For the first 12 participants in Experiment 1 (including two excluded participants), stimuli were presented on a 32-inch Cambridge Research Systems Display++ LCD monitor with 1920 × 1080-pixel resolution, hardware gamma correction, and a refresh rate of 120 Hz. For the remainder of participants in Experiment 1 and all participants in Experiment 2, stimuli were presented on a 24-inch Asus VG428QE 3D monitor with 1920 × 1080-pixel resolution and a refresh rate of 120 Hz. This change was made because we moved labs during this project owing to equipment availability constraints and had no consistent effect on participant performance (see Supplementary Figure S1). A gamma correction of 2 was applied to the Asus display.

### Experiment 1

Twenty-three participants completed Experiment 1. Upon visual inspection of individual data, three participants performed at chance in the 0° separation condition and were thus excluded from all other analyses. Each participant completed 1320 trials. All participants were shown the same set of stimuli, which comprised of 1320 unique reference/target source images and 1320 unique foil source images which were pseudorandomly selected from a bank of 9,361 potential images. Images were selected pseudorandomly to ensure unique source images were used for every stimulus, with no overlap in image content (per the source image generation technique used, described in Section 3.3., Stimuli), and no replacement after image selection. Trial order was randomized for each participant. Participants also completed 20 practice trials before starting the experiment, using randomly selected images not used in the experiment trials. On each trial, participants were shown a fixation point for 700 ms, followed by the reference patch for 500 ms, a random pink noise mask for 1000 ms and, finally, the target and foil patches (Figure 2A). Target and foil patches were presented until participants indicated which one came from the same broader image as the reference. Importantly, participants were not told about the spatial separation manipulations used to generate the stimuli. Participants completed all trials in a single testing session split into 12 blocks, with self-paced breaks between each block.

### Experiment 2

#### Stimuli

**Figure 3.**
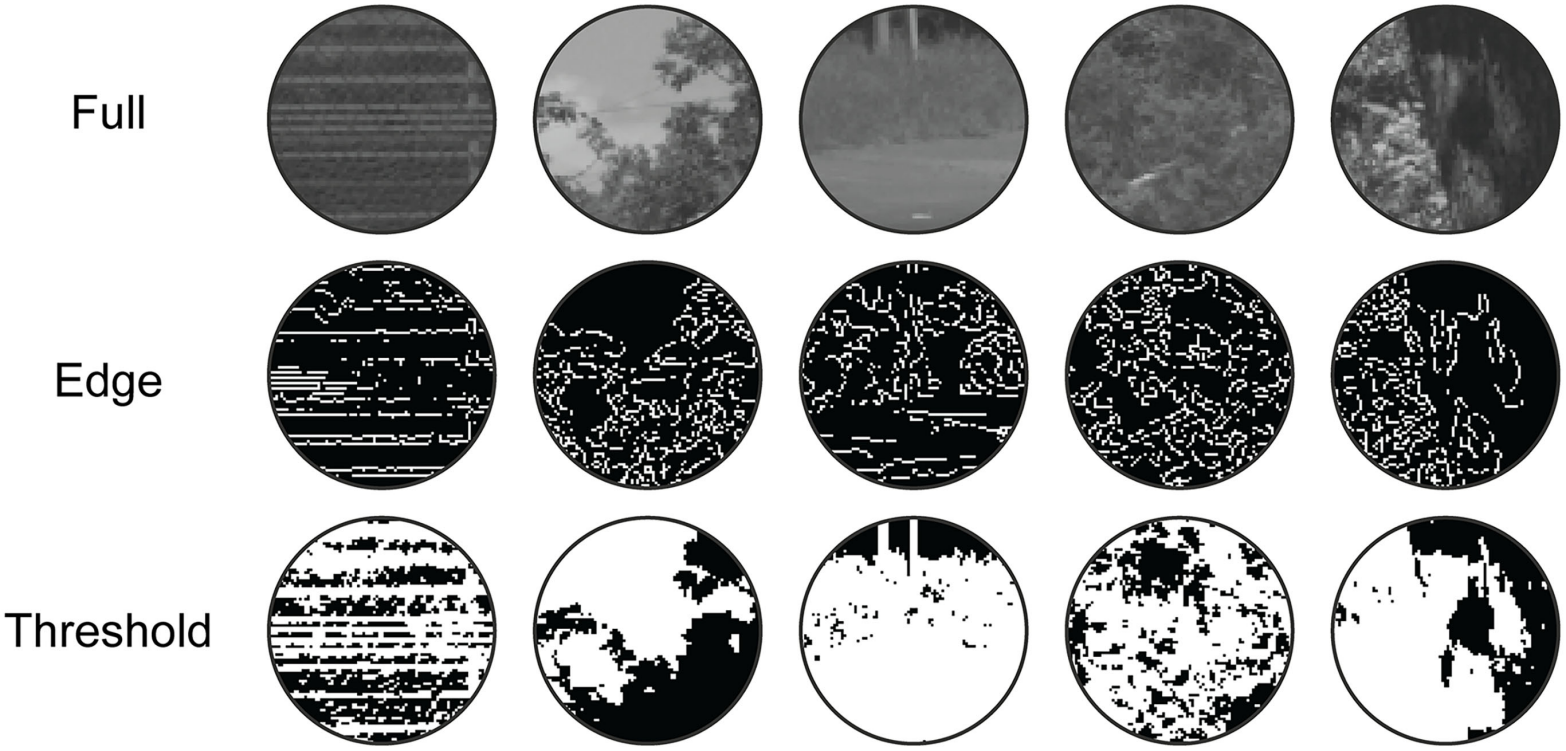
Example stimuli from each image processing condition in Experiment 2. Each row represents a different condition (i.e., full, edge, and threshold), with the same five source stimuli used for each row. *Note:* These conditions were only applied/compared for Experiment 2. Experiment 1 used full images only.

To further investigate specific low-level feature contributions to scene region associations, Experiment 2 introduced an additional image manipulation. This new manipulation had three conditions, adapted from processing techniques used in Viengkham and Spehar (2022; Figure 3). In the “full” patch condition, participants were shown unaltered foil/target patches (beyond the general processing outlined in Section 3.3., Stimuli). Hence, apart from the number of trials, the full patch condition was identical to Experiment 1. In the “threshold” patch condition, the median pixel value of the individual foil and test patches was calculated, and pixel values above this were set to white and pixel values below/equal to this were set to black. Finally, for the “edge” patch condition, source images were reduced to their edge content using MATLAB's edge() function (a basic edge detector function) using the ‘Canny’ edge-finding method. Patches were then cropped from the resulting images. In the current experiment, such image processing was only applied to the test images, and not the reference images—which were the same across conditions (i.e., the reference image was “full” regardless of condition). By leaving the reference image unaltered, we investigated whether participants could relate the image statistics in full unaltered image regions to subsequent deteriorated test images.

#### Design

Participants (N = 20; no exclusions) completed 1386 trials. All participants were shown the same set of stimuli, with the same stimuli also being used for each of the three image processing conditions. Hence, the stimulus set comprised of 462 unique reference/target source images and 462 unique foil source images, which were pseudorandomly selected from a bank of 9,361 potential images. Again, images were selected pseudorandomly to ensure unique source images were used for every stimulus, with no overlap in image content (per the source image generation technique used, described in Section 3.3., Stimuli), and no replacement after image selection. Trial order was randomized for each participant. Participants completed 20 practice trials before starting the experiment, using randomly selected images not used in actual experiment trials. On each trial, participants were shown a fixation point for 700 ms, followed by the reference patch for 500 ms, a random pink noise mask for 200 ms and, finally, were shown the target and foil patches (Figure 2A). Target and foil patches were presented until a response was made with a button press, indicating which patch participants believed came from the same broader image as the reference. Again, participants were not made aware of the spatial separation manipulations used to generate the stimuli. Participants completed all trials in a single testing session split into 14 blocks, with self-paced breaks between each block.

### Analyses

Aside from the generalized linear multilevel model (GLMM) (below), other inferential statistics were Bayesian analyses conducted in JASP, using response accuracy as the dependent variable.

#### GLMM

We aimed to identify a descriptive model that could test whether measured image statistics are sufficient for emulating human responses—as such, the current model was not intended to simulate the same processes performed by the brain. We therefore modelled participants’ perceptual decisions under a GLMM framework, fit to all participants’ data in each experiment. The predictors are image statistics that are intended as approximate descriptors of the image features that we think are relevant for perception, and so our specific model aims to redescribe observers’ performance in terms of those metrics. In this way, we investigate the extent to which human responses rely on information associated with such simple metrics.

The use of a GLMM framework allowed us to match the range of responses from the model to the range of responses from human observers. The GLMM was implemented using MATLAB's fitglme() function, which fits a generalized linear mixed effects model (defined by fixed and random effects, analogous to the lmer() function in the lme4 package in R) using maximum pseudo-likelihood estimation. Participants’ responses were coded as 0 or 1, to indicate incorrect and correct responses, respectively. The model was provided with predictors based on the same stimuli participants saw across both conditions—comprising of only full patches for Experiment 1, or combinations of full/edge/threshold patches for Experiment 2. Predictors were based on stimuli provided as square images, with the circularly windowed stimulus patch flanked by uniform mid-grey. Like participants, the model was not given access to information about the image-separation (in other words, separation distance and azimuth were not entered as predictors). Instead, the model implemented image similarity metric predictors, as such metrics have been shown to be useful for predicting other human perceptual decisions relating to naturalistic images (A-Izzeddin et al., 2024; Rideaux et al., 2022; Sebastian et al., 2017; Sebastian et al., 2020). More specifically, we included two image statistic predictors based on low-level (i.e., image computable) information in the model: pixel-wise luminance root mean square (RMS) error and phase-invariant structural similarity, each of which is described below. We predicted the probability of a correct response as:

(1) $$\begin{array}{c} P\;\left(\mathrm{correct}\right)\;=\;\mathrm{logistic}\left(\eta \right),\; \end{array}$$

where logistic represents the inverse of the logit link-function, and:

(2) $$\begin{array}{c} \eta =\beta_{0}+\beta_{1}I_{\Delta }+\beta_{2}S_{\Delta }+\beta_{3}I_{\Delta }S_{\Delta },\; \end{array}$$

where β_0_ is the intercept term, β_1_ is the weight of the pixel-wise luminance difference, *I*_Δ_, β_2_ is the weight of phase-invariant structural similarity, *S*_Δ_, and β_3_ is the weight of the interaction *I*_Δ_*S*_Δ_. *I*_Δ_ and *S*_Δ_ are defined below. We implemented this model as a GLMM to partially pool coefficient estimates across observers and images, both of which were random effects in the model. The random effect of images refers to the unique image combination on a given trial. We describe this approach in greater detail in Rideaux et al. (2022).

Our first predictor—pixel-wise luminance error—involved computing the RMS error between the reference patch and the target patch, as well as between the reference patch and the foil patch (see Figure 5A and Figure 8 for visualization of these relationships across experiments). We then determined which patch was most similar to the reference by calculating the difference score between these error scores, subtracting the foil luminance error from the target luminance error, giving *I*_Δ_. Positive values of *I*_Δ_ indicate that the foil patch was more similar to the reference (in terms of pixel distances), whereas negative values of *I*_Δ_ indicate that the target patch was more similar to the reference. We then log-scaled absolute *I*_Δ_ values, in line with logarithmic scaling performed by the visual system (e.g., Weber's law), and multiplied them by their original sign. Finally, to improve the interpretability of beta coefficients, we standardized the predictor values by dividing each value of *I*_Δ_ by the standard deviation of all values of *I*_Δ_ (Schielzeth, 2010). The resulting beta weights can be thereby interpreted as proportional to a change of the corresponding predictor by 1 standard deviation. We note here that *I*_Δ_ values were never 0, given these represent differences in the target and foil patches’ similarity scores. Hence, *I*_Δ_ could only be 0 if both the foil and target had the same similarity scores—this is only likely to occur should the foil and target be identical, which was never the case in the current study.

Our second predictor was phase-invariant structural similarity. A phase-invariant measure of structural similarity was included owing to the likelihood of substantial luminance changes across a photo (e.g., some areas might be in direct sunlight and others in shade). Such variations in luminance could lead to very low pixel-wise luminance correlations, despite the underlying structure remaining the same. Hence, the phase-invariant structural similarity predictor quantified the spatial similarity of the patches’ spatial frequency and orientation content, while ignoring the absolute luminance values of the pixels (Sebastian et al., 2017). Per Sebastian et al. (2017), we first found the amplitude spectrum of the reference (A*_S_*), target (A*_T_*), and foil (A*_F_*) patches: for each patch, amplitude spectra were calculated by subtracting the mean of the patch, applying a fast Fourier transform, and taking the absolute complex value. To quantify the structural similarity between the reference and the target (targetSS), as well as between the reference and the foil (foilSS), we took the cosine of the vector angle between the amplitude spectra of the relevant test patch (see Figure 5, Supplementary Figures S11, and S12 for visualization of this relationship across experiments):

(3) $$\begin{array}{c} \mathrm{targetSS}\;=\;\frac{A_{S}\cdot A_{T}}{∥A_{S}∥\;∥A_{T}∥}\; \end{array}$$

(4) $$\begin{array}{c} \mathrm{foilSS}\;=\frac{A_{S}\cdot A_{F}}{∥A_{S}∥\;∥A_{F}∥}\; \end{array}$$

We then determined which patch was most similar to the reference by calculating the difference score between these structural similarity scores, subtracting foilSS from targetSS, giving *S*_Δ_. Positive values of *S*_Δ_ indicate that the target patch was more similar to the reference (in terms of structural similarity), whereas negative values of *S*_Δ_ indicate that the foil patch was more similar to the reference. We finally log-scaled absolute *S*_Δ_ values, multiplied them by their original sign, and standardized by dividing each value of *S*_Δ_ by the standard deviation of all values of *S*_Δ_.

We note that, in Experiment 2, one trial in the edge-only condition resulted in target and foil images that were both devoid of any edges (i.e., two black patches were presented). As such, both patches yielded the same similarity scores for both predictors, leading to a difference score of 0, which resulted in an infinite value after undergoing log-scaling. We therefore removed trials with this combination of patches (across all image filtering conditions) from analyses—this was a total of three trials per participant, because each participant saw the same set of images, and each image filtering condition repeated the same source set of images. Otherwise, identical model architecture was implemented for both Experiments 1 and 2, with the only difference between experiments being the input predictor values, which were based on the specific stimuli used in each experiment respectively.

**Figure 4.**
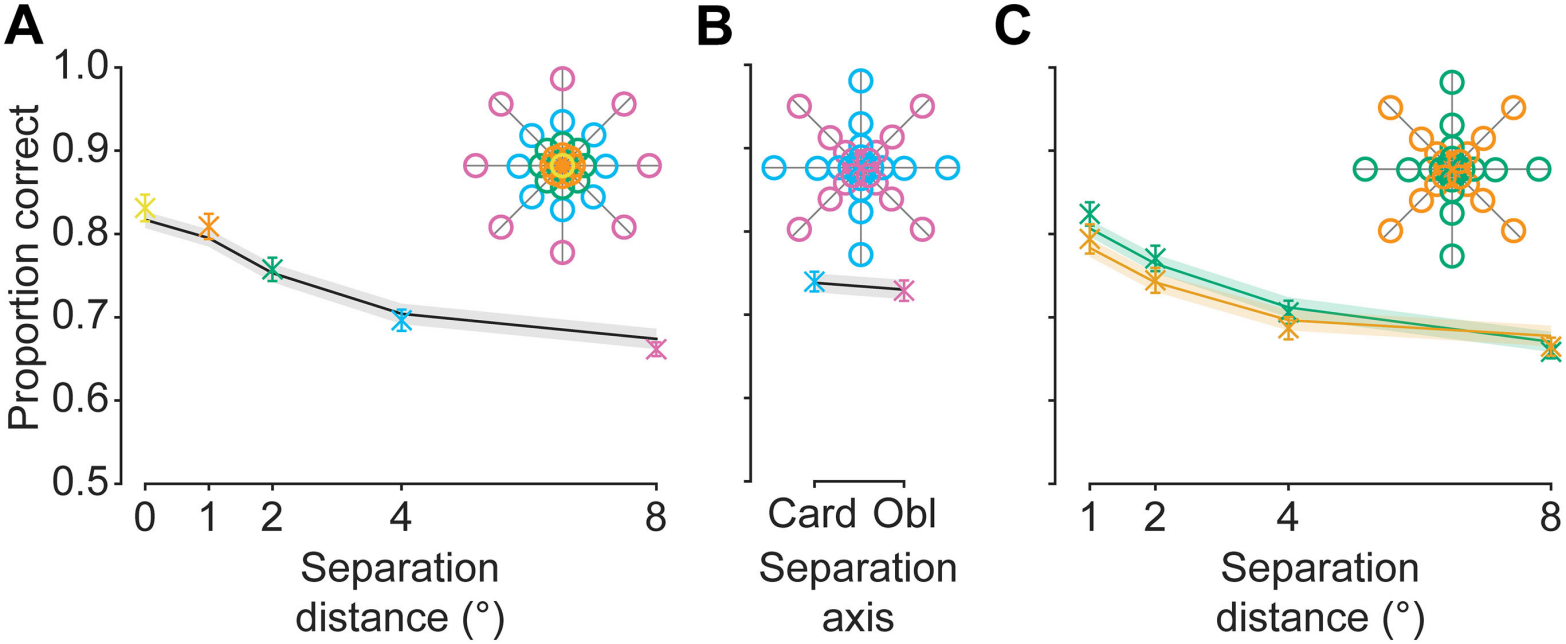
Experiment 1 behavioral and GLMM results. (**A**) Effect of separation distance (*x* axis) on the proportion of correct responses (*y* axis). Data points are color coded and are averaged across spatial locations of the same color in the legend (inset). Solid line represents GLMM predictions based on luminance and structural similarity heuristics between the target and reference patch vs. the foil and reference patch. (**B**) Effect of separation axis (cardinal vs. oblique) on the proportion of correct responses. (**C**) The interaction between separation distance (*x* axis) and separation axis (separate lines; see legend) on the proportion of correct responses. Error bars: ±1 SEM for participant responses (in some cases, standard errors are smaller than the point size). Shaded regions: ±1 SEM for trial-by-trial model predictions.

To visualize the model fits (e.g., Figures 4, 6, and 7, solid lines), we generated predicted response probabilities for each trial. Predictions were yielded from the GLMM fit output, ‘F,’ using ‘F.predict.’ Note that, in generating trial-by-trial predictions, we were able to partial predictions into separation distance/azimuth conditions with associated standard error of the mean estimates. To reiterate, however, separations were not used as model predictors.

We implemented an alternative GLMM that was identical to the current model described; however, we used log ratio scores instead of difference scores. Specifically, we calculated both pixel-wise luminance error and phase-invariant structural similarity scores as in the previous model comparing the target and foil with the reference. For each predictor, we then divided the target's similarity score by the foil's and took the log of this value. To avoid infinite values, we added a constant value of 10^−^^6^ to similarity scores before taking the log ratio. This was particularly relevant in the 0° separation condition, where the pixel-wise luminance error for the target is 0. We found highly similar fits for this model as compared with the difference score model across both experiments, and therefore only report difference score model results.

We compared the performance of the full model (as described) to alternative difference score models containing only the phase-invariant structural similarity main effect, or both the phase-invariant structural similarity and pixel-wise luminance difference main effects. Nested models were formally compared, with full output and comparison results presented in the supplemental materials for both Experiment 1 (Supplementary Tables S1–S4) and Experiment 2 (Supplementary Tables S7–S10). In addition, we compared the performance of the full model to an alternative model that used mean luminance (calculated by comparing the mean pixel values of the stimuli) and overall image contrast (calculated by comparing the standard deviations of pixel values of the stimuli) differences between stimuli as predictors for both experiments. We found this alternative model to only out-perform the full model described for Experiment 2 data (Supplementary Tables S11–S12), but not for Experiment 1 data (Supplementary Tables S5–S6). Although we note that the full model was not the best-performing model in all comparisons, in the main body of the current text, we report the full model results for consistency.

Finally, to further investigate the performance of our GLMM parameterization across experiments, we ran a pooled data GLMM. Specifically, data from both Experiment 1 and Experiment 2 were collated in a single dataset and run through the same GLMM architecture as previously specified: predictors derived from pixel-wise luminance RMS error and phase-invariant structural similarity, with observers and images used as random effects in the model.

## Results

### Experiment 1: Can similarity judgments be predicted from low-level features?

In Experiment 1, participants were presented with two image patches (a target and a foil) and selected which they judged to have been drawn from the same broader image/scene as a preceding reference patch (Figure 2A). The spatial location that the target patch was drawn from relative to the reference was manipulated via distance (0°, 1°, 2°, 4°, or 8° of visual angle) and azimuth (0°, 45°, 90°, 135°, 180°, 225°, 270°, or 315°) (Figure 2B), but participants did not have knowledge of this spatial information. To assess performance, the proportion of correct responses (i.e., correctly identifying the target patch) was calculated for each condition. Based on known pixel-wise correlations between image regions and how they change with separation, we expected participants’ performance to decline with increasing separation distance, and that participants would perform better for cardinal separation axes than for obliques (Field, 1987; Frazor & Geisler, 2006; Simoncelli & Olshausen, 2001). We first describe the qualitative patterns of the data, and then describe the quantitative GLMM fits.

Behavioral data, as shown in Figure 4, were inspected by a 2 (separation axis: cardinal, oblique) × 4 (separation distance: 1°, 2°, 4°, 8°; 0° was not included as this condition could not interact with separation axis) Bayesian repeated measures ANOVA. As expected, participants’ performance decreased as the separation distance increased between the reference and the target, supported by extreme evidence in favor of an overall separation distance main effect (BF_10_ = 9.662 × 10^38^) and with post hoc comparisons in favor of a difference in task performance between each individual separation distance condition (minimum BF_10_ = 893.056). Counter to expectations, however, there was no clear effect of separation axis on performance (BF_10_ = 0.528), with comparable accuracy levels for cardinal and oblique offsets (see Supplementary Figure S1 for individual data). Visually, there appears to be an interaction effect between the separation axis and distance. Specifically, there appeared to be a benefit for cardinal offsets at the smallest distances that diminished in magnitude with increasing separation distance. This result pattern would suggest that information carried along the cardinal axes is only noticeably useful (beyond that observed for oblique axes) up to a certain separation distance, after which information conveyed by either separation axis is equivocal. However, there was equivocal evidence in favor of the alternative and null hypotheses in relation to the interaction (BF_10_ = 1.083). As such, there was no clear effect of the separation axis on task performance.

When provided with two identical target patches (i.e., the 0° separation condition), participants’ response accuracy was worse than what would be expected if their performance in this condition depended only on a simple lapse rate. It is possible that this result is due to a proportion of memory-related errors and response errors, both of which should be distributed uniformly across separation conditions. Importantly, all participants show a very similar pattern of result across all separation distances, but with substantial differences in absolute performance across observers (see Supplementary Figure S1 for individual data). We further note that there was greater between-subject variance for the 0° separation condition as compared with the other separation distances (see Supplementary Figure S2). As such, we tentatively suggest that the interaction between individual participant performance, as well as the increased variance observed for the 0°, were the primary contributors to the unexpectedly low performance observed for the 0° separation condition.

**Figure 5.**
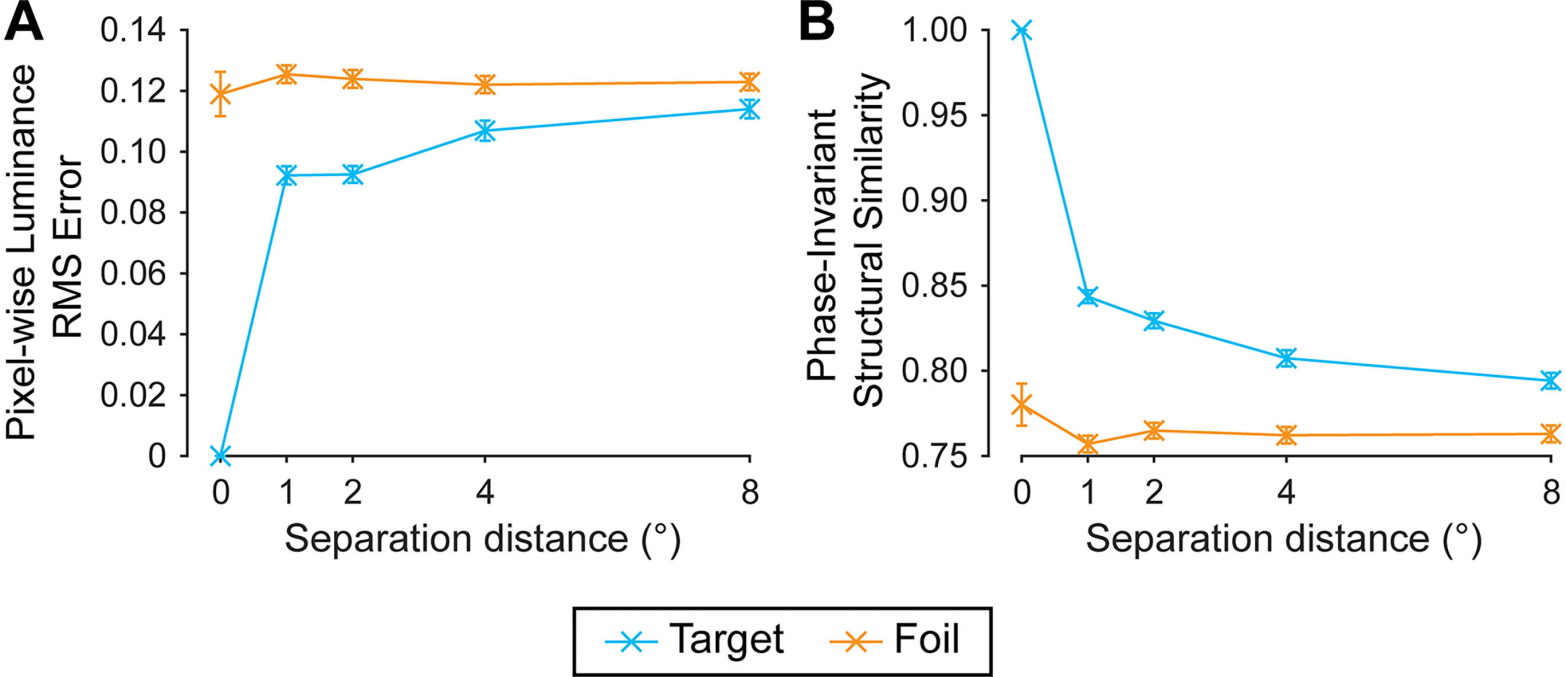
Effect of separation distance on image statistic predictors for Experiment 1. (**A**) Mean effect of separation distance on pixel-wise luminance RMS error values, comparing the reference patch with the target (blue) and foil (orange). Note: Higher values indicate lower levels of similarity with the reference patch. (**B**) Mean effect of separation distance on phase-invariant structural similarity values, comparing the reference patch with the target and foil. Note: Higher values indicate higher levels of similarity with the reference patch. These statistics were used as predictors of observers’ reports (see Section 3.7.2., GLMM, for more detail). Error bars: ±1 SEM (in some cases, standard errors are smaller than the point size). See Supplementary Figure S3 for effect of separation azimuth.

A GLMM was fit to the data to quantify performance using low-level, or image-computable, information. Predictors were similarity metrics based on pixel-wise luminance and phase-invariant structure correlations. Raw pixel-wise luminance and phase-invariant structural similarity scores and their interactions with separation conditions were inspected independent of their use as predictors in the GLMM. Specifically, as separation distance increased, there were clear increases in target/reference pixel-wise luminance RMS error and decreases in phase-invariant structural similarity (Figure 5, blue lines). Together, these relationships verify that the target patch is most similar to the reference patch for smaller versus larger separation distances, corroborating previous work (Field, 1987; Frazor & Geisler, 2006; Harrison, 2021; Simoncelli & Olshausen, 2001). In contrast, raw pixel-wise luminance and phase-invariant structural similarity scores for the foil/reference did not vary systematically with separation distance (Figure 5, orange). Further, across all separation distances, the foil was less similar overall to the reference than was the target. This consistently greater similarity of the target than the foil fits well with participants’ high proportion of correct responses across conditions. In sum, we observed systematic effects of the separation manipulations on similarity, verifying the effectiveness of the manipulation of low-level feature correlations via spatial separation.

Using predictors based on low-level information, we observed a consistent relationship between the GLMM predictions and participants’ responses (Figure 4, solid lines). Indeed, both pixel-wise luminance and phase-invariant structural similarity were significant predictors in the model (maximum *p* < 0.001); however, the interaction between predictors was not significant (*p* = 0.983) (see Supplementary Table S1 for full model output). Importantly, the model did not include predictors for separation conditions (i.e., distance and azimuth). Nevertheless, after generating predictions, the data were partialled into separation conditions; they revealed high consistency with behavioral data for each individual condition, including at 0° separation (Figure 4). Overall, the high level of congruence observed between the model and the behavioral data suggests observer responses use low-level features, or highly correlated alternative features. Indeed, our findings suggest that complex, higher-level features may not be necessary to perform such perceptual judgments for naturalistic stimuli.

For the sake of completeness, several other analyses were performed to explore the effects of individual separation azimuths, as well as separation fields (e.g., upper vs. lower separations relative to horizontal) (see Supplementary Figures S4 and S5). The GLMM fit all exploratory separation classifications well. Additional inspections will not be discussed in depth because they largely did not reveal clear effects. One exception was the observation of a slightly better performance for left separations as compared with right, relative to vertical (see Supplementary Figure S5). This separation field effect is most likely due to a stimulus set-specific bias that made similarity judgments marginally easier for left separations compared with right separations. The presence of a stimulus bias is supported by the GLMM predictions, which closely corresponded with this response pattern.

### Experiment 2: Which low-level features facilitate similarity judgments?

On finding that low-level information correlations can predict participants’ image region associations in Experiment 1, Experiment 2 sought to more directly investigate which features facilitate participants’ performance. The same image region association task as in Experiment 1 was implemented, but now the target/foil images were passed through one of three image processing manipulations: “full,” “threshold,” and “edge” processing (Figure 3). By thresholding the images or reducing them to their edge content, we reduce the complexity of our stimuli to only a subset of image features that participants may use to facilitate their responses. By thresholding the images, we highlight the distribution of high- and low-luminance regions across the image. Alternatively, by reducing images to their edges, we instead highlight the structural content of the images. All other separation distance and azimuth conditions from Experiment 1 were repeated in Experiment 2, and observers’ responses were again modelled using low-level information predictors.

**Figure 6.**
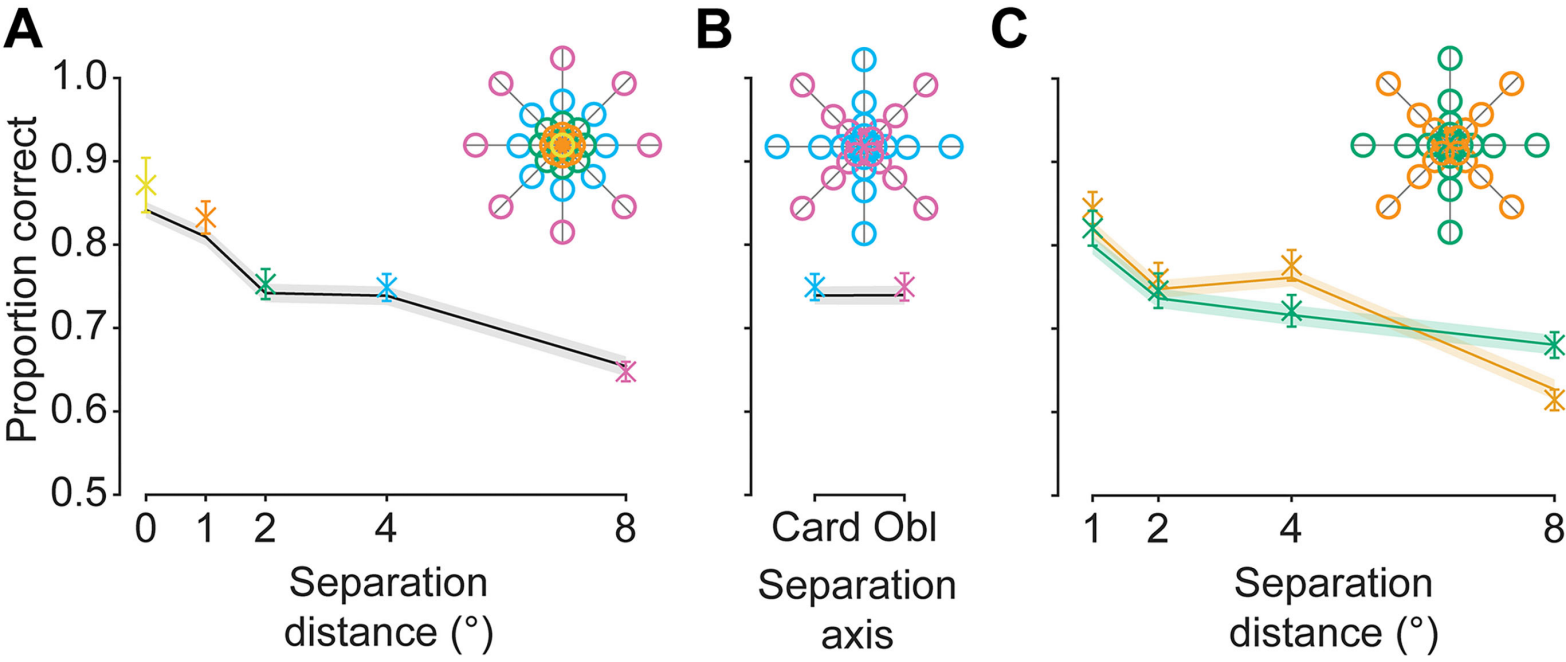
Experiment 2 behavioral and GLMM results for the full image condition only. (**A**) Effect of separation distance (*x* axis) on the proportion of correct responses (*y* axis). Data points are color coded and are averaged across spatial locations of the same color in the legend (inset). Solid line represents GLMM predictions based on luminance and structural similarity heuristics between the target and reference patch vs. the foil and reference patch. (**B**) Effect of separation axis (cardinal vs. oblique) on the proportion of correct responses. (**C**) Interaction between separation distance (*x* axis) and separation axis (separate lines; see legend) on the proportion of correct responses. Error bars: ±1 SEM for participant responses (in some cases, standard errors are smaller than the point size). Shaded regions: ±1 SEM for trial-by-trial model predictions.

We explored the impact of separation distance, separation axis, and their interaction on participants’ performance for Experiment 2 for the full image condition to compare patterns of results with those found in Experiment 1 (Figure 6; see Supplementary Figures S7–S9 for other image processing conditions). An initial Bayesian 3 (image processing: full, edge, threshold) × 2 (separation axis: cardinal, oblique) × 4 (separation distance: 1°, 2°, 4°, and 8°; 0° was not included because this condition could not interact with the separation axis) repeated measures ANOVA was conducted and revealed moderate evidence in favor of there being no three-way interaction (BF_10_ = 0.200). Given the lack of three-way interaction and that the purposes of describing behavioral effects is to compare with the patterns observed in Experiment 1, for simplicity, we report the results of a Bayesian 2 (separation axis: cardinal, oblique) × 4 (separation distance: 1°, 2°, 4°, and 8°) repeated measures ANOVA for the full image condition only.

As shown in Figure 6, increasing separation distance between the reference and the target led to decreased performance in the full image condition, as in Experiment 1, supported by extreme evidence in favor of a separation distance main effect (BF_10_ = 3.295 × 10^26^). Post hoc comparisons demonstrated extreme evidence in favor of a difference between all separation distance conditions (minimum BF_10_ = 2.118 × 10^5^) with the exception of the comparison of the 2° and 4° separation distance conditions, which revealed moderate evidence in favor of there being no difference in performance (BF_10_ = 0.180). The equivocal performance in the 2° and 4° separation distance conditions (see Figure 6A) is likely due to a bias in the stimulus set used for Experiment 2. Specifically, given the random selection of source images, the features participants used to complete the task likely vary between conditions. In this case, it is likely that such features in the 2° and 4° separation distance conditions happened to result in similar difficulty levels. Low accuracy scores were observed for the 0° separation distance condition. We suspect this result, as in Experiment 1, is likely due to a combination of the overall variability in participant-to-participant performance (see Supplementary Figure S6) and the greater response variance for this datapoint (see Supplementary Figure S9). There was moderate evidence in favor of no effect of separation axis on performance (BF_10_ = 0.189), with comparable accuracy levels for cardinal and oblique offsets, consistent with the lack of clear separation axis effect found in Experiment 1 (Figure 6B). Extreme evidence was found in favor of an interaction effect between separation axis and separation distance (BF = 1.014 × 10^4^) (Figure 6C), with a very different qualitative appearance of response patterns as compared with Experiment 1 (where there was no evidence in favor of an interaction; comparing Figures 4C with 6C). This result, as with the dip in performance for the 2° separation distance condition, likely also followed from a bias in the features present in the current stimulus set. The results of Experiment 2, although largely consistent with those of Experiment 1, therefore suggest that the outcomes are influenced by the specific stimulus set used whose features (e.g., low-level pixel-wise information correlations) influence association judgments differentially across separation conditions. We performed several other exploratory analyses, as in Experiment 1, which we do not discuss in depth here (but see Supplementary Figures S6–S10 for additional inspections, as well as individual data).

**Figure 7.**
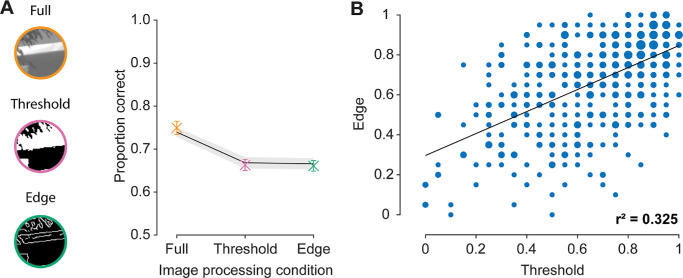
Experiment 2 stimulus examples and results. (**A**) Effect of image processing condition (*x* axis) on the proportion of correct responses (*y* axis). Data points are color coded according to image conditions depicted to the left of the plot. Solid line represents GLMM predictions based on luminance and structural similarity heuristics between the target and reference patch vs. the foil and reference patch. Error bars: ±1 SEM for participant responses. Shaded regions: ±1 SEM for trial-by-trial model predictions. (**B**) Correlation between the average response for each threshold image (*x* axis) with their corresponding (i.e., generated from the same source image) edge image (*y* axis). Values of 1 indicate that the target was always chosen over the foil, and values of 0 indicate the foil was always chosen over the target. Note: There are overlapping data points, indicated by larger symbols.

In Experiment 2, participants made similarity judgments on either full, threshold, or edge-only images. Participants performed best in the full patch condition across all separation conditions (Figure 7A; see Supplementary Figures S6–S8 and S10 for individual data and interaction with other separation manipulations), suggesting neither of the image filtering techniques produced stimuli that contained sufficient information to yield the same levels of performance. The initial Bayesian 3 (image processing: full, edge, threshold) × 2 (separation axis: cardinal, oblique) × 4 (separation distance: 1°, 2°, 4°, and 8°) repeated measures ANOVA revealed extreme evidence in favor of an image processing condition main effect (BF_10_ = 4.135 × 10^21^). Here, post hoc comparisons revealed extreme evidence in favor of higher response accuracy in the full image condition as compared with the threshold (BF_10_ = 4.183 × 10^25^) and edge conditions (BF_10_ = 2.114 × 10^24^). Further, there was strong evidence in favor of there being no difference in performance between the edge and threshold patch conditions (BF_10_ = 0.088). This was supported by a significant positive correlation between the average response to edge vs. threshold versions of each target image (*r^2^* = 0.325; *p* < 0.001) (Figure 7B). These results suggest that the edge and threshold conditions were similarly informative, and that participants potentially used additional features beyond pixel-wise luminance or structural information to perform the task when provided with full images. Alternatively, poorer performance for the threshold and edge image conditions might have followed from low-level information correlations being reduced by the image processing techniques used.

**Figure 8.**
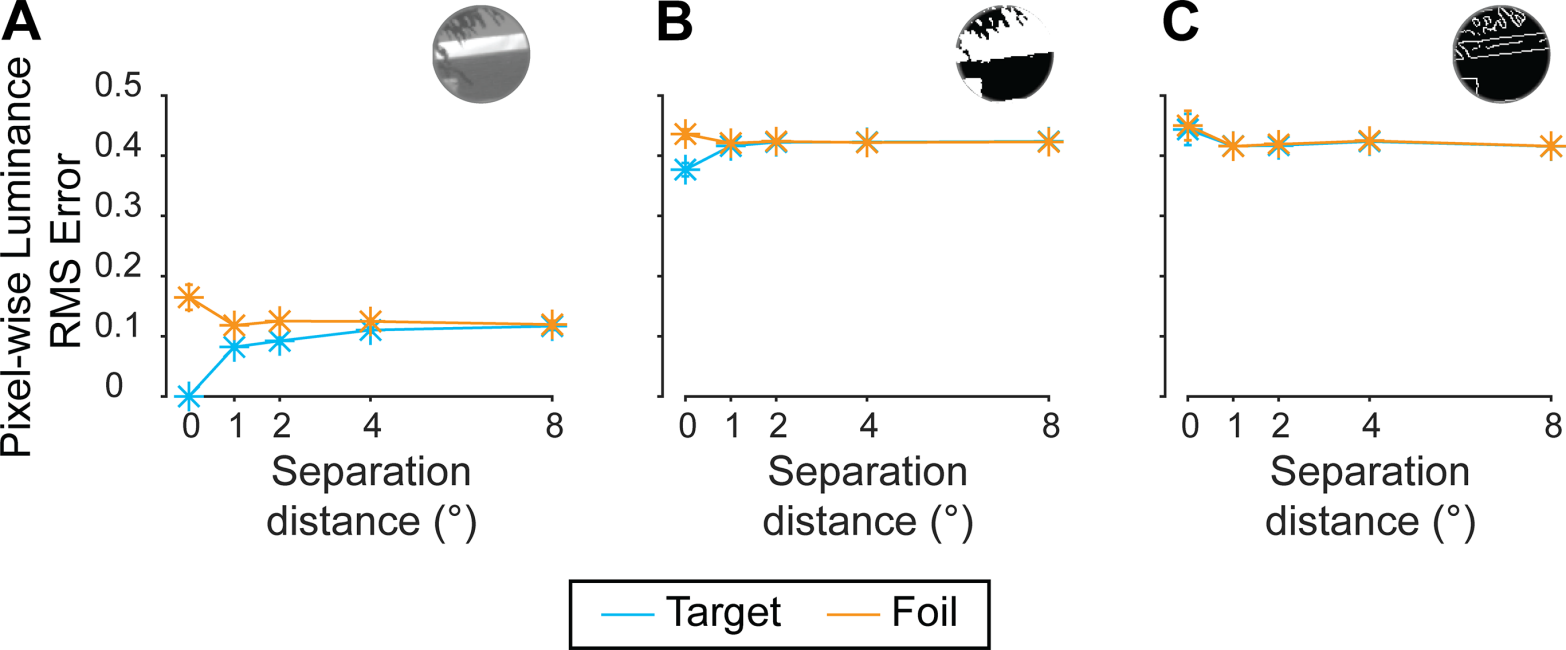
Effect of separation distance on pixel-wise luminance RMS error values for Experiment 2. (A) Mean effect of separation distance on pixel-wise luminance RMS error values, comparing the reference patch with the target (blue) and foil (orange) in the full image condition. (B) Mean effect of separation distance on pixel-wise luminance RMS error values, comparing the reference patch with the target and foil in the threshold image condition. (C) Mean effect of separation distance on pixel-wise luminance RMS error values, comparing the reference patch with the target and foil in the edge image condition. These statistics were used as predictors of observers’ reports (see Section 3.7.2., GLMM, for more detail). Error bars: ±1 SEM (in some cases, standard errors are smaller than the point size). See Supplementary Figures S11 and S12 for overall effect of separation distance, effects of separation azimuth, as well as phase-invariant structural similarity results.

To investigate the impact of image processing conditions on low-level pixel-wise information correlations, raw pixel-wise luminance and phase-invariant structural similarity scores were inspected. Such scores were computed for patches after having undergone any relevant image processing for a given trial. Indeed, target patches were less similar to the reference after undergoing the edge and threshold processing techniques. Taking pixel-wise luminance as an example, for the full image condition, a similar pattern and similar RMS error values were found to those reported for in Experiment 1 (comparing Figures 5A with 8A). Specifically, as the separation distance increased, there was an increase in the pixel-wise luminance RMS error (indicating decreasing similarity) for the target, with a relatively stable error for the foil across separation distances (Figure 8A). However, we note the similar levels of RMS error between the 2° and 4° conditions for both targets and foils, in line with similar levels of response accuracy. Further, this relationship was much less clear for the threshold and edge image conditions, with far greater overlap in target and foil RMS error scores across separation distance (Figures 8B and 8C), as well as higher RMS error values overall (comparing Figures 8B and 8C with 8A). Together, these results indicate that the processing of the patches led to significant disruptions to the pixel information, resulting in less informative low-level information correlations. These lower correlations are consistent with the lower levels of performance observed across the threshold and edge image conditions as compared with the full image condition.

Pixel-wise luminance and phase-invariant structural similarity scores were used as predictors for a GLMM that was fit to Experiment 2 data, as in Experiment 1. As observed in Experiment 1, phase-invariant structural similarity was a significant predictor (*p* = 0.012) and the interaction between predictors was not significant (*p* = 0.327). However, unlike in Experiment 1, pixel-wise luminance error was not a significant predictor (*p* = 0.382), consistent with the larger degree of overlap in raw pixel-wise error scores as a result of image processing (Figures 8B and 8C), suggesting that this predictor was unable to distinguish between the target and foil (see Supplementary Table S7 for full model output) in Experiment 2 stimuli. Nonetheless, across all conditions, there was consistency between model predictions and behavioral data, including at 0° separation. Model/behavioral data consistency across conditions supports the notion that stimulus set-specific biases can influence low-level feature correlations and participant responses. Specifically, the equivocal performance in the 2° and 4° separation distance conditions, as well as the interaction between separation axis and distance, were both accounted for by GLMM predictions (Figure 6). Further, GLMM predictions were consistent with behavioral performance for each image processing condition, suggesting lower levels of performance for edge and threshold images can also be accounted for by changes in low-level information correlations (Figure 7A, solid line). When we pool observations across both Experiment 1 and Experiment 2 and apply the same GLMM framework, we find both pixel-wise luminance error and phase-invariant structural similarity to be significant predictors (*p* < 0.001 for both), but not their interaction (*p* = 0.308) (see Supplementary Table S13 for full model output). Hence, the results of Experiment 2 and from the pooling of data across experiments further demonstrate the explanatory power for participant responses of features that are, or are highly correlated with, low-level features—in particular structural similarity. Conversely, our findings suggest that increasingly complex information may not be necessary to explain naturalistic scene region association judgments.

## Discussion

We investigated observers’ capacity to associate naturalistic scene regions and the predictability of such responses from low-level visual features, rather than complex features. Across two experiments, participants viewed image patches, windowed from broader naturalistic images, and indicated which two of three were drawn from the same broader scene, allowing us to measure participants’ ability to accurately identify which regions came from the same scene. Critically, we also implemented a GLMM framework to predict responses using low-level (i.e., image-computable) information. Specifically, the predictors for the model were pixel-wise luminance and phase-invariant structural similarity. Using a GLMM framework allowed an assessment of the potential contribution of such low-level information to image region associations. The GLMM was able to account for participants’ responses, suggesting that low-level information, or features highly correlated with such information is sufficient to facilitate image region associations without the need to rely on complex features.

### Observers can associate isolated naturalistic image regions

We found evidence that observers are able to associate isolated image regions with one another, building on existing evidence for the computational capacity to perform such associations (Field, 1987; Frazor & Geisler, 2006; Harrison, 2021; Simoncelli & Olshausen, 2001). Importantly, spatial separations between the two patches that did belong to the same broader scene were manipulated across experiments—namely, via distance and azimuth manipulations—to introduce systematic variance in low-level feature correlations. In both Experiment 1 and Experiment 2, participants were unaware of the spatial manipulations used, but consistently performed above chance at all separation distances and axes, suggesting our ability to associate regions of space is robust to such spatial offsets.

The results of the current study are consistent with the broader scene perception literature, which has demonstrated that observers can process scene information rapidly and with a high degree of fidelity. For example, observers can perform scene categorization judgments with presentation times as short as a couple of hundred milliseconds (Fei-Fei, Iyer, Koch, & Perona, 2007; Thorpe, Fize, & Marlot, 1996; VanRullen & Thorpe, 2001). Unlike past studies that provided participants with whole naturalistic images that conveyed entire scenes, the current study used smaller windowed image regions, allowing us to effectively limit the amount of information available in a given image to approximate that of the fovea (A-Izzeddin et al., 2024). For example, in most cases, the windowing of image regions almost completely eliminated the presence of multiple objects that could have indicated the regions’ spatial position within the scene. By providing such limited scene information, the current study expands on previous work, demonstrating that we require very little information to understand properties of the broader scene. Previous research suggests that the presence of such location-based object relationships facilitates perceptual tasks using naturalistic scene stimuli, such as object-based visual search (Brockmole et al., 2006; Brockmole & Henderson, 2006b; Brockmole & Henderson, 2006a; Eckstein et al., 2006; Loftus & Mackworth, 1978). However, the current study suggests that such relationships at the object level may not be necessary for scene mapping, with this potentially requiring only very limited visual information.

### Judgments on complex stimuli are reducible to low-level feature correlations

Our results suggest that image region associations can be accounted for using low-level feature correlations. We found that the GLMM provides an excellent fit to participant responses using low-level information correlations as predictors. Indeed, our model does not include an extensive number of predictors, and other potential candidate models could include alternative or additional low-level features, as well as more complex features. However, we present the current model, having provided predictions that are consistent with observed behavioral responses, as a suitable benchmark against which other models can be tested in future. Methods for calculating low-level feature correlations between naturalistic image regions have been established in the literature, demonstrating clear computational capacity for performing such region associations (Field, 1987; Frazor & Geisler, 2006; Harrison, 2021; Simoncelli & Olshausen, 2001). Further, in the contour grouping literature, there is evidence to suggest that contour association judgments largely follow from basic co-occurring image statistics (Elder & Goldberg, 2002; Geisler, Perry, Super, & Gallogly, 2001; Geisler & Perry, 2009). The current study is consistent and extends on this previous computation and contour grouping work, demonstrating that scene region associations are similarly well-accounted for by low-level features.

We have a demonstrated capacity to perceive and make perceptual decisions using low-level features. For example, we know that observers are sensitive to and are able to reproduce individual orientations (Appelle, 1972; Bays, 2014; Berkley et al., 1975; Campbell et al., 1966; Dakin, 2001; de Gardelle et al., 2010; Emsley, 1925; Girshick et al., 2011; Harrison & Bays, 2018; Henderson & Hollingworth, 1999; Pratte et al., 2016; Taylor & Bays, 2018; Westheimer & Beard, 1998) and are even sensitive to the average of an array of orientations (Dakin et al., 2009; Dakin & Watt, 1997; Parkes, Lund, Angelucci, Solomon, & Morgan, 2001). Each of these judgments requires the effective processing of available low-level information. The current study extends on this work by investigating the perception of such low-level information when it is embedded within more complex stimuli. Indeed, we have found evidence to suggest that judgments on naturalistic image regions can be accounted for by low-level feature correlations alone.

### The role of alternative visual information

In Experiment 2, participants were provided with stimuli that were either unaltered or had undergone processing to reduce information to either thresholded contrast or edge content. Here, perhaps unsurprisingly, participants’ performance dropped substantially when given limited image information in comparison with when given unaltered image regions. Low-level image-computable information correlations between the reference and target patches were heavily impacted by the edge and threshold image processing techniques implemented. Specifically, image processing resulted in much lower similarity scores, resembling scores found when comparing the foil and reference patches, suggesting that poorer performance is to be expected if participants are using low-level information. However, there remains the possibility that alternative information was being used to support the higher level of performance observed for unaltered images that is eliminated by further image processing.

Another clear candidate for alternative information participants may use in the full image condition is higher-level or semantic information. By higher-level information, we refer to features that convey meaning and are not image computable, such as the arrangement of chairs in a room that denote a living room rather than a dining room (Neri, 2014). A-Izzeddin et al. (2024) had participants infer the upright orientation of randomly oriented image regions. In that study, the same stimulus generation method was used, cropping the same sized image regions from the same bank of photographs. As part of their study, A-Izzeddin et al. (2024) conducted a control experiment and found that only a very small proportion of stimuli (∼4%) generated using this method conveyed informative high-level information that could disambiguate the patches for the task used. Further, they found that, when participants were given patches with informative content, responses were varied and sometimes yielded large errors in orientation judgments. Hence, even when provided with such informative content, participants do not seem able to effectively use such information to inform their judgments. The results of A-Izzeddin et al. (2024) thus suggest that high-level information is unlikely to be a substantial contributor to participants’ performance in the current task when given unaltered image regions. It, therefore, follows that any further removal of high-level information from the images after undergoing processing is unlikely to explain the observed drop in performance.

Importantly, the GLMM used in Experiment 1 and Experiment 2 could predict responses using low-level information, across the image processing conditions used. The GLMM was also able to predict responses across all separation conditions, despite not having access to this spatial information. The ability to predict responses using such low-level information suggests that, even if participants use higher-level visual information to perform the task, we can still account for judgments using the most basic of visual features. There are, of course, additional visual features that participants might use and that were not accounted for in the current model. Such features, similar to those inspected in the current model, likely interacted with the application of image processing techniques in Experiment 2, which may have driven the poorer performance observed. Future studies could investigate the impact of manipulating other features beyond pixel-wise luminance- and edge-defined structure on image association judgments.

Indeed, the notion that different features are associated with observer responses depending on image manipulations is supported by our finding that an alternative model using mean luminance and RMS contrast outperformed the current model for Experiment 2, but not for Experiment 1. One simple interpretation is that the image manipulations in Experiment 2 necessitated the use of different image features than for Experiment 1. For example, it makes less sense for an observer to compare pixel-wise luminance values when images have been thresholded (e.g., Figure 3). In such a case where pixel values are binarized, an alternative metric such as mean luminance is likely more useful for this task. Further manipulations of such alternative features therefore pose an opportunity to advance our understanding of which features participants actively prioritize when performing image region association judgments. However, the performance of the current model suggests that any relevant alternative features follow from, or are highly correlated with, the current image-computable information used as predictors, leading to our ability to account for participants’ responses to unaltered image regions.

### Associating regions of space informing complex scene mapping

Beyond the association of two regions of space, there has been continued speculation surrounding the mechanism by which observers generate a coherent representation of a viewed scene (Berens et al., 2021; Chen et al., 2023; Cohen et al., 1999; Goh et al., 2004; Hassabis & Maguire, 2007; Robertson et al., 2016; Singer & Gray, 1995; Steel et al., 2021; Treisman, 1998). The efficiency with which we process scene information necessitates that we be highly adept at this process to enable coherent representations of the scene we are viewing on behaviorally relevant timescales. Further work is needed to more directly address the specific mechanisms underlying broader scene mapping, as well as the features most useful in making such associations. The current study takes a modest step in this direction by showing that we are indeed able to make prompted scene association judgments based on limited available information. In particular, we found that these associations can be accounted for by low-level feature correlations, suggesting that such features are strongly associated with those underlying judgments made for complex stimuli. Our findings therefore suggest high-level information may not be necessary for completing perceptual judgments related to complex, naturalistic stimuli.

## Supplementary Material

Supplement 1

## References

[bib1] A-Izzeddin, E. J., Mattingley, J. B., & Harrison, W. J. (2024). The influence of natural image statistics on upright orientation judgements. *Cognition,* 242, 105631, 10.1016/j.cognition.2023.105631. 37820487

[bib2] Akbas, E., & Eckstein, M. P. (2017). Object detection through search with a foveated visual system. *PLoS Computational Biology,* 13(10), e1005743, 10.1371/journal.pcbi.1005743. 28991906 PMC5669499

[bib3] Aks, D. J., & Sprott, J. C. (1996). Quantifying aesthetic preference for chaotic patterns. *Empirical Studies of the Arts,* 14(1), 1–16, 10.2190/6V31-7M9R-T9L5-CDG9.

[bib4] Appelle, S. (1972). Perception and discrimination as a function of stimulus orientation: The ‘oblique effect’ in man and animals. *Psychological Bulletin,* 78(4), 266–278, 10.1037/h0033117. 4562947

[bib5] Bar, M. (2004). Visual objects in context. *Nature Reviews Neuroscience,* 5(8), 8, 10.1038/nrn1476. 15263892

[bib6] Bar, M., & Ullman, S. (1993). Spatial context in recognition. *Perception,* 25, 324–352. 10.1068/p250343 8804097

[bib7] Bays, P. M. (2014). Noise in neural populations accounts for errors in working memory. *Journal of Neuroscience,* 34(10), 3632–3645, 10.1523/JNEUROSCI.3204-13.2014. 24599462 PMC3942580

[bib8] Berens, S. C., Joensen, B. H., & Horner, A. J. (2021). Tracking the emergence of location-based spatial representations in human scene-selective cortex. *Journal of Cognitive Neuroscience,* 33(3), 445–462, 10.1162/jocn_a_01654. 33284080 PMC8658499

[bib9] Berkley, M. A., Kitterle, F., & Watkins, D. W. (1975). Grating visibility as a function of orientation and retinal eccentricity. *Vision Research,* 15(2), 239–244, 10.1016/0042-6989(75)90213-8. 1129981

[bib10] Bex, P. J. (2010). (In) Sensitivity to spatial distortion in natural scenes. *Journal of Vision,* 10(2), 23, 10.1167/10.2.23. PMC2924673 20462324

[bib11] Bex, P. J., Solomon, S. G., & Dakin, S. C. (2009). Contrast sensitivity in natural scenes depends on edge as well as spatial frequency structure. *Journal of Vision,* 9(10), 1–19, 10.1167/9.10.1. 19810782 PMC3612947

[bib12] Biederman, I., Mezzanotte, R. J., & Rabinowitz, J. C. (1982). Scene perception: Detecting and judging objects undergoing relational violations. *Cognitive Psychology,* 14(2), 143–177, 10.1016/0010-0285(82)90007-X. 7083801

[bib13] Brainard, D. H. (1997). The Psychophysics Toolbox. *Spatial Vision,* 10(4), 433–436, 10.1163/156856897X00357. 9176952

[bib14] Brockmole, J. R., Castelhano, M. S., & Henderson, J. M. (2006). Contextual cueing in naturalistic scenes: Global and local contexts. *Journal of Experimental Psychology: Learning, Memory, and Cognition,* 32(4), 699–706, 10.1037/0278-7393.32.4.699. 16822141

[bib15] Brockmole, J. R., & Henderson, J. M. (2006a). Recognition and attention guidance during contextual cueing in real-world scenes: Evidence from eye movements. *Quarterly Journal of Experimental Psychology,* 59(7), 1177–1187, 10.1080/17470210600665996. 16769618

[bib16] Brockmole, J. R., & Henderson, J. M. (2006b). Using real-world scenes as contextual cues for search. *Visual Cognition,* 13(1), 99–108, 10.1080/13506280500165188.

[bib17] Burge, J., & Geisler, W. S. (2011). Optimal defocus estimation in individual natural images. *Proceedings of the National Academy of Sciences of the United State of America,* 108(40), 16849–16854, 10.1073/pnas.1108491108. PMC3189032 21930897

[bib18] Campbell, F. W., Kulikowski, J. J., & Levinson, J. (1966). The effect of orientation on the visual resolution of gratings. *Journal of Physiology,* 187(2), 427–436, 10.1113/jphysiol.1966.sp008100. 5972182 PMC1395930

[bib19] Carandini, M. (2005). Do we know what the early visual system does? *Journal of Neuroscience,* 25(46), 10577–10597, 10.1523/JNEUROSCI.3726-05.2005. 16291931 PMC6725861

[bib20] Chen, L., Cichy, R. M., & Kaiser, D. (2023). Alpha-frequency feedback to early visual cortex orchestrates coherent natural vision (p. 2023.02.10.527986). bioRxiv, 10.1101/2023.02.10.527986. PMC10637741 37948520

[bib21] Cohen, N. J., Ryan, J., Hunt, C., Romine, L., Wszalek, T., & Nash, C. (1999). Hippocampal system and declarative (relational) memory: Summarizing the data from functional neuroimaging studies. *Hippocampus,* 9(1), 83–98, 10.1002/(SICI)1098-1063(1999)9:1<83::AID-HIPO9>3.0.CO;2-7. 10088903

[bib22] Coppola, D. M., Purves, H. R., McCoy, A. N., & Purves, D. (1998). The distribution of oriented contours in the real world. *Proceedings of the National Academy of Sciences of the United State of America,* 95(7), 4002–4006, 10.1073/pnas.95.7.4002. PMC19952 9520482

[bib23] Dakin, S. C. (2001). Information limit on the spatial integration of local orientation signals. *Journal of the Optical Society of America,* 18(5), 1016–1026, 10.1364/JOSAA.18.001016. 11336204

[bib24] Dakin, S. C., Bex, P. J., Cass, J. R., & Watt, R. J. (2009). Dissociable effects of attention and crowding on orientation averaging. *Journal of Vision,* 9(11), 1–16, 10.1167/9.11.28. 20053091 PMC2927104

[bib25] Dakin, S. C., & Watt, R. J. (1997). The computation of orientation statistics from visual texture. *Vision Research,* 37(22), 3181–3192, 10.1016/S0042-6989(97)00133-8. 9463699

[bib26] Davenport, J. L., & Potter, M. C. (2004). Scene consistency in object and background perception. *Psychological Science,* 15(8), 559–564, 10.1111/j.0956-7976.2004.00719.x. 15271002

[bib27] David, S. V., Vinje, W. E., & Gallant, J. L. (2004). Natural stimulus statistics alter the receptive field structure of V1 neurons. *Journal of Neuroscience,* 24(31), 6991–7006, 10.1523/JNEUROSCI.1422-04.2004. 15295035 PMC6729594

[bib28] de Gardelle, V., Kouider, S., & Sackur, J. (2010). An oblique illusion modulated by visibility: Non-monotonic sensory integration in orientation processing. *Journal of Vision,* 10(10), 1–9, 10.1167/10.10.6. 20884471

[bib29] Eckstein, M. P., Drescher, B. A., & Shimozaki, S. S. (2006). Attentional cues in real scenes, saccadic targeting, and Bayesian priors. *Psychological Science,* 17(11), 973–980, 10.1111/j.1467-9280.2006.01815.x. 17176430

[bib30] Elder, J. H., & Goldberg, R. M. (2002). Ecological statistics of Gestalt laws for the perceptual organization of contours. *Journal of Vision,* 2(4), 5, 10.1167/2.4.5. 12678582

[bib31] Emsley, H. H. (1925). Irregular astigmatism of the eye: Effect of correcting lenses. *Transactions of the Optical Society,* 27(1), 28–42, 10.1088/1475-4878/27/1/304.

[bib32] Essock, E. A., DeFord, J. K., Hansen, B. C., & Sinai, M. J. (2003). Oblique stimuli are seen best (not worst!) in naturalistic broad-band stimuli: A horizontal effect. *Vision Research,* 43(12), 1329–1335, 10.1016/S0042-6989(03)00142-1. 12742103

[bib33] Fei-Fei, L., Iyer, A., Koch, C., & Perona, P. (2007). What do we perceive in a glance of a real-world scene? *Journal of Vision,* 7(1), 10, 10.1167/7.1.10. 17461678

[bib34] Field, D. J. (1987). Relations between the statistics of natural images and the response properties of cortical cells. *Journal of the Optical Society of America A,* 4(12), 2379, 10.1364/JOSAA.4.002379. 3430225

[bib35] Frazor, R. A., & Geisler, W. S. (2006). Local luminance and contrast in natural images. *Vision Research,* 46(10), 1585–1598, 10.1016/j.visres.2005.06.038. 16403546

[bib36] Friedman, A. (1979). Framing pictures: The role of knowledge in automatized encoding and memory for gist. *Journal of Experimental Psychology: General,* 108, 316–355, 10.1037/0096-3445.108.3.316. 528908

[bib37] Geisler, W. S., & Perry, J. S. (2009). Contour statistics in natural images: Grouping across occlusions. *Visual Neuroscience,* 26(1), 109–121, 10.1017/S0952523808080875. 19216819 PMC2660385

[bib38] Geisler, W. S., Perry, J. S., Super, B. J., & Gallogly, D. P. (2001). Edge co-occurrence in natural images predicts contour grouping performance. *Vision Research,* 41(6), 711–724, 10.1016/S0042-6989(00)00277-7. 11248261

[bib39] Girshick, A. R., Landy, M. S., & Simoncelli, E. P. (2011). Cardinal rules: Visual orientation perception reflects knowledge of environmental statistics. *Nature Neuroscience,* 14(7), 926–932, 10.1038/nn.2831. 21642976 PMC3125404

[bib40] Goh, J. O. S., Siong, S. C., Park, D., Gutchess, A., Hebrank, A., & Chee, M. W. L. (2004). Cortical areas involved in object, background, and object-background processing revealed with functional magnetic resonance adaptation. *Journal of Neuroscience,* 24(45), 10223–10228, 10.1523/JNEUROSCI.3373-04.2004. 15537894 PMC6730187

[bib41] Hansen, B. C., & Essock, E. A. (2004). A horizontal bias in human visual processing of orientation and its correspondence to the structural components of natural scenes. *Journal of Vision,* 4(12), 1044–1060, 10.1167/4.12.5. 15669910

[bib42] Hansen, B. C., Essock, E. A., Zheng, Y., & Deford, J. K. (2003). Perceptual anisotropies in visual processing and their relation to natural image statistics. *Network: Computation in Neural Systems,* 14(3), 501–526, 10.1088/0954-898X_14_3_307. 12938769

[bib43] Harrison, W. J. (2021). Luminance and contrast of images in the THINGS database. bioRxiv. 2021.07.08.451706, 10.1101/2021.07.08.451706. 35296165

[bib44] Harrison, W. J., & Bays, P. M. (2018). Visual working memory is independent of the cortical spacing between memoranda. *Journal of Neuroscience,* 38(12), 3116–3123, 10.1523/JNEUROSCI.2645-17.2017. 29459370 PMC5864153

[bib45] Harrison, W. J., Stead, I., Wallis, T. S. A., Bex, P. J., & Mattingley, J. B. (2024). A computational account of transsaccadic attentional allocation based on visual gain fields. *Proceedings of the National Academy of Sciences of the United State of America,* 121(27), e2316608121, 10.1073/pnas.2316608121. PMC11228487 38941277

[bib46] Hassabis, D., & Maguire, E. A. (2007). Deconstructing episodic memory with construction. *Trends in Cognitive Sciences,* 11(7), 299–306, 10.1016/j.tics.2007.05.001. 17548229

[bib47] Henderson, J. M. (2003). Human gaze control during real-world scene perception. *Trends in Cognitive Sciences,* 7(11), 498–504, 10.1016/j.tics.2003.09.006. 14585447

[bib48] Henderson, J. M., & Hollingworth, A. (1999). High-level scene perception. *Annual Review of Psychology,* 50(1), 243–271, 10.1146/annurev.psych.50.1.243. 10074679

[bib49] Henderson, J. M., Weeks, P. A. Jr., & Hollingworth, A. (1999). The effects of semantic consistency on eye movements during complex scene viewing. *Journal of Experimental Psychology: Human Perception and Performance,* 25, 210–228, 10.1037/0096-1523.25.1.210.

[bib50] Hidalgo-Sotelo, B., Oliva, A., & Torralba, A. (2005). Human learning of contextual priors for object search: Where does the time go? *2005 IEEE Computer Society Conference on Computer Vision and Pattern Recognition (CVPR’05) - Workshops,* 86–86. San Diego, CA, 10.1109/CVPR.2005.470.

[bib51] Hollingworth, A., & Henderson, J. M. (2000). Semantic informativeness mediates the detection of changes in natural scenes. *Visual Cognition,* 7(1–3), 213–235, 10.1080/135062800394775.

[bib52] Keil, M. S., & Cristóbal, G. (2000). Separating the chaff from the wheat: Possible origins of the oblique effect. *Journal of the Optical Society of America A,* 17(4), 697, 10.1364/JOSAA.17.000697. 10757177

[bib53] Knill, D., Field, D., & Kersten, D. (1990). Human discrimination of fractal images1. *Journal of the Optical Society of America A-Optics Image Science and Vision,* 7, 1113–1123, 10.1364/JOSAA.7.001113. 2362228

[bib54] Loftus, G. R., & Mackworth, N. H. (1978). Cognitive determinants of fixation location during picture viewing. *Journal of Experimental Psychology: Human Perception and Performance,* 4, 565–572, 10.1037/0096-1523.4.4.565. 722248

[bib55] Mirza, M. B., Adams, R. A., Mathys, C. D., & Friston, K. J. (2016). Scene construction, visual foraging, and active inference. *Frontiers in Computational Neuroscience,* 10, 56, https://www.frontiersin.org/articles/10.3389/fncom.2016.00056. 27378899 10.3389/fncom.2016.00056 PMC4906014

[bib56] Neider, M. B., & Zelinsky, G. J. (2006). Scene context guides eye movements during visual search. *Vision Research,* 46(5), 614–621, 10.1016/j.visres.2005.08.025. 16236336

[bib57] Neri, P. (2014). Semantic control of feature extraction from natural scenes. *Journal of Neuroscience,* 34(6), 2374–2388, 10.1523/JNEUROSCI.1755-13.2014. 24501376 PMC3913878

[bib58] Noton, D., & Stark, L. (1971). Scanpaths in eye movements during pattern perception. *Science,* 171(3968), 308–311. 5538847 10.1126/science.171.3968.308

[bib59] Olshausen, B. A., & Field, D. J. (2005). How close are we to understanding V1? *Neural Computation,* 17(8), 1665–1699, 10.1162/0899766054026639. 15969914

[bib60] Palmer, S. E. (1975). The effects of contextual scenes on the identification of objects. *Memory & Cognition,* 3(5), 519–526, 10.3758/BF03197524. 24203874

[bib61] Parkes, L., Lund, J., Angelucci, A., Solomon, J. A., & Morgan, M. (2001). Compulsory averaging of crowded orientation signals in human vision. *Nature Neuroscience,* 4(7), 7, 10.1038/89532. 11426231

[bib62] Pelli, D. G. (1997). The VideoToolbox software for visual psychophysics: Transforming numbers into movies. *Spatial Vision,* 10(4), 437–442, 10.1163/156856897X00366. 9176953

[bib63] Pratte, M. S., Park, Y. E., Rademaker, R. L., & Tong, F. (2016). Accounting for stimulus-specific variation in precision reveals a discrete capacity limit in visual working memory. *Journal of Experimental Psychology: Human Perception and Performance,* 43(1), 6–17, 10.1037/xhp0000302. PMC5189913 28004957

[bib64] Rideaux, R., West, R. K., Wallis, T. S. A., Bex, P. J., Mattingley, J. B., & Harrison, W. J. (2022). Spatial structure, phase, and the contrast of natural images. *Journal of Vision,* 22(1), 4, 10.1167/jov.22.1.4. PMC8762697 35006237

[bib65] Robertson, C. E., Hermann, K. L., Mynick, A., Kravitz, D. J., & Kanwisher, N. (2016). Neural representations integrate the current field of view with the remembered 360° panorama in scene-selective cortex. *Current Biology,* 26(18), 2463–2468, 10.1016/j.cub.2016.07.002. 27618266

[bib66] Schielzeth, H. (2010). Simple means to improve the interpretability of regression coefficients. *Methods in Ecology and Evolution,* 1(2), 103–113, 10.1111/j.2041-210X.2010.00012.x.

[bib67] Sebastian, S., Abrams, J., & Geisler, W. S. (2017). Constrained sampling experiments reveal principles of detection in natural scenes. *Proceedings of the National Academy of Sciences of the United State of America,* 114(28), E5731–E5740, 10.1073/pnas.1619487114. PMC5514707 28652323

[bib68] Sebastian, S., Seemiller, E. S., & Geisler, W. S. (2020). Local reliability weighting explains identification of partially masked objects in natural images. *Proceedings of the National Academy of Sciences of the United State of America,* 117(47), 29363–29370, 10.1073/pnas.1912331117. PMC7703648 33229552

[bib69] Series, P., & Seitz, A. (2013). Learning what to expect (in visual perception). *Frontiers in Human Neuroscience,* 7, 668, 10.3389/fnhum.2013.00668. 24187536 PMC3807544

[bib70] Simoncelli, E. P., & Olshausen, B. A. (2001). Natural image statistics and neural representation. *Annual Review of Neuroscience,* 24(1), 1193–1216, 10.1146/annurev.neuro.24.1.1193. 11520932

[bib71] Singer, W., & Gray, C. M. (1995). Visual feature integration and the temporal correlation hypothesis. *Annual Review of Neuroscience,* 18(1), 555–586, 10.1146/annurev.ne.18.030195.003011. 7605074

[bib72] Sonkusare, S., Breakspear, M., & Guo, C. (2019). Naturalistic stimuli in neuroscience: Critically acclaimed. *Trends in Cognitive Sciences,* 23(8), 699–714, 10.1016/j.tics.2019.05.004. 31257145

[bib73] Spehar, B., Clifford, C. W. G., Newell, B. R., & Taylor, R. P. (2003). Universal aesthetic of fractals. *Computers & Graphics,* 27(5), 813–820, 10.1016/S0097-8493(03)00154-7.

[bib74] Spehar, B., & Taylor, R. P. (2013). Fractals in art and nature: Why do we like them? *Human Vision and Electronic Imaging XVIII,* 8651, 298–309, 10.1117/12.2012076.

[bib75] Sprott, J. C. (1993). Automatic generation of strange attractors. *Computers & Graphics,* 17(3), 325–332, 10.1016/0097-8493(93)90082-K.

[bib76] Steel, A., Billings, M. M., Silson, E. H., & Robertson, C. E. (2021). A network linking scene perception and spatial memory systems in posterior cerebral cortex. *Nature Communications,* 12(1), 1, 10.1038/s41467-021-22848-z. PMC8113503 33976141

[bib77] Summerfield, C., & Egner, T. (2009). Expectation (and attention) in visual cognition. *Trends in Cognitive Sciences,* 13(9), 403–409, 10.1016/j.tics.2009.06.003. 19716752

[bib78] Taylor, R., & Bays, P. M. (2018). Efficient coding in visual working memory accounts for stimulus-specific variations in recall. *Journal of Neuroscience,* 38(32), 7132–7142, 10.1523/JNEUROSCI.1018-18.2018. 30006363 PMC6083451

[bib79] Taylor, R., Spehar, B., Hagerhall, C., & Van Donkelaar, P. (2011). Perceptual and physiological responses to Jackson Pollock's fractals. *Frontiers in Human Neuroscience,* 5, 60, https://www.frontiersin.org/articles/10.3389/fnhum.2011.00060. 21734876 10.3389/fnhum.2011.00060 PMC3124832

[bib80] Thorpe, S., Fize, D., & Marlot, C. (1996). Speed of processing in the human visual system. *Nature,* 381, 520–522. 8632824 10.1038/381520a0

[bib81] Torralba, A., & Oliva, A. (2003). Statistics of natural image categories. *Network: Computation in Neural Systems,* 14(3), 391–412, 10.1088/0954-898X_14_3_302. 12938764

[bib82] Treisman, A. (1998). Feature binding, attention and object perception. *Philosophical Transactions of the Royal Society B: Biological Sciences,* 353(1373), 1295–1306. 10.1098/rstb.1998.0284 PMC1692340 9770223

[bib83] VanRullen, R., & Thorpe, S. J. (2001). The time course of visual processing: From early perception to decision-making. *Journal of Cognitive Neuroscience,* 13(4), 454–461, 10.1162/08989290152001880. 11388919

[bib84] Viengkham, C., Isherwood, Z., & Spehar, B. (2022). Fractal-scaling properties as aesthetic primitives in vision and touch. *Axiomathes,* 32(5), 869–888, 10.1007/s10516-019-09444-z.

[bib85] Viengkham, C., & Spehar, B. (2018). Preference for fractal-scaling properties across synthetic noise images and artworks. *Frontiers in Psychology,* 9, 1439, https://www.frontiersin.org/articles/10.3389/fpsyg.2018.01439. 30210380 10.3389/fpsyg.2018.01439 PMC6123544

[bib86] Viengkham, C., & Spehar, B. (2022). Beyond visual aesthetics: The role of fractal-scaling characteristics across the senses. *Journal of Perceptual Imaging,* 5, 000406-1–000406-14, 10.2352/J.Percept.Imaging.2021.4.3.030406, https://search.library.uq.edu.au.

[bib87] Wallis, T. S. A., & Bex, P. J. (2012). Image correlates of crowding in natural scenes. *Journal of Vision,* 12(7), 1–19, 10.1167/12.7.6. PMC4503217 22798053

[bib88] Westheimer, G., & Beard, B. L. (1998). Orientation dependency for foveal line stimuli: Detection and intensity discrimination, resolution, orientation discrimination and Vernier acuity. *Vision Research,* 38(8), 1097–1103, 10.1016/S0042-6989(97)00248-4. 9666969

