# Supplementary material for: Low-level features predict perceived similarity for naturalistic images
Source: J Vis. 2025 Oct 7;25(12):11. doi: 10.1167/jov.25.12.11 (PMC12514980; doi:10.1167/jov.25.12.11)
Supplement: Supplement 1 [file jovi-25-12-11_s001.pdf]

Low-level features predict perceived similarity for naturalistic images

Emily J A-Izzeddin[1,2,3*], Thomas SA Wallis[3,4], Jason B Mattingley[2,5], William J Harrison[5,6]

[1] Department of Experimental Psychology, Justus Liebig University Giessen, Giessen, Germany

[2] Queensland Brain Institute, University of Queensland, St Lucia, Queensland, Australia

[3] Center for Mind, Brain and Behavior (CMBB), University of Marburg and Justus Liebig University Giessen

[4] Institute of Psychology & Centre for Cognitive Science, Technical University of Darmstadt, Darmstadt, Germany

[5] School of Psychology, University of Queensland, St Lucia, Queensland, Australia

[6] School of Health, University of the Sunshine Coast, Sippy Downs, Queensland 4556 Australia

* Corresponding author

Email: Emily.A-Izzeddin@psychol.uni-giessen.de

Address: Justus Liebig University Giessen, Department of Experimental Psychology

Otto-Behaghel-Str. 10F

35394 Gießen, Germany

S.1. Experiment 1 individual data

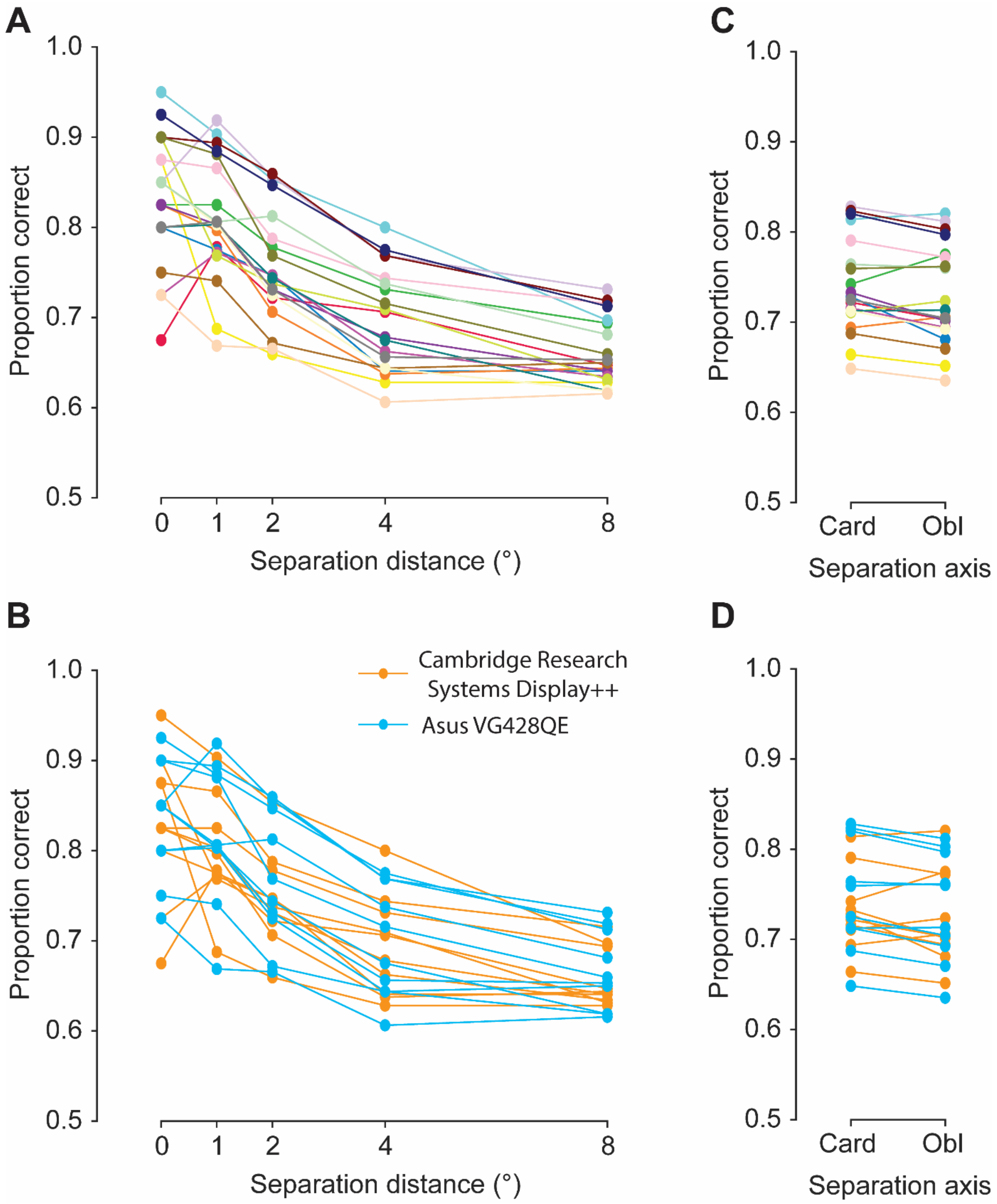

Figure S.1. Individual data for Experiment 1. A) Individual data corresponding to **Figure 4A**, showing the effect of separation distance (x-axis) on the proportion of correct responses (y-axis). **B)** The same data as in Panel A, but colour-coded to show participants who participated with the two different monitor set-ups (see inset legend) as outlined in **Section 3.4., Apparatus**. We see no consistent effect of monitor. **C)** Individual data corresponding to **Figure 4B**, showing the effect of separation axis (cardinal vs oblique) on the proportion of correct responses. **D)** The same data as in Panel C, but colour-coded to show participants who participated with the two different monitor set-ups (see legend) as outlined in **Section 3.4., Apparatus**. We see no consistent effect of monitor. Solid lines are used to connect individual participants' datapoints and do not represent model fits.

S.2. Experiment 1 variance data

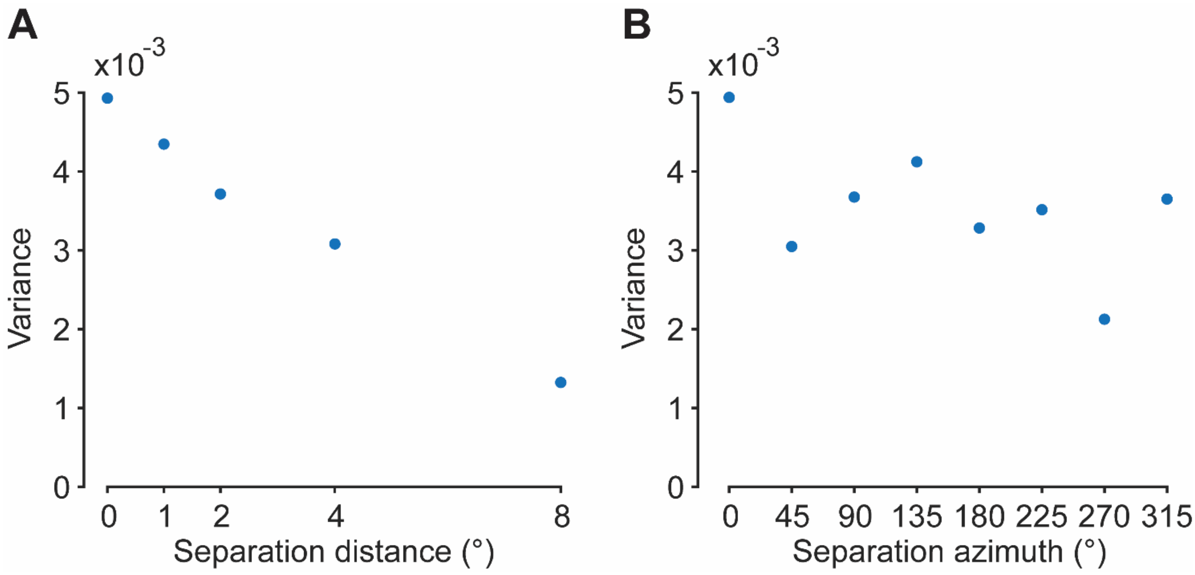

Figure S.2. Experiment 1 variance data corresponding to response accuracy data presented in Figure 4A and Figure S.4A. The effect of separation distance (**A**; x-axis) and azimuth (**B**; x-axis) on response variance (y-axis). Variance was calculated using MATLAB's var() function, finding the variance in participants' proportion of correct responses at each separation distance/azimuth condition.

S.3. Effect of separation azimuth on Experiment 1 GLMM predictor values

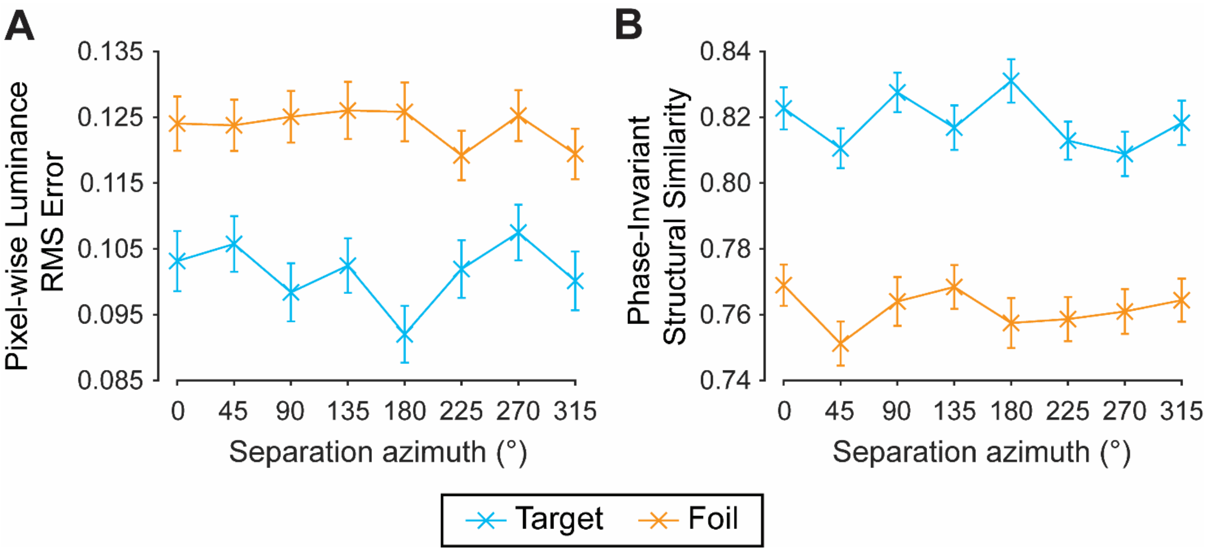

Figure S.3. Effect of separation azimuth on predictor values implemented for Experiment 1's GLMM. A) Mean effect of separation azimuth on pixel-wise luminance RMS error values, comparing the reference patch with the target (blue) and foil (orange). **B)** Mean effect of separation azimuth on phase-invariant structural similarity values, comparing the reference patch with the target and foil. Error bars: ±1 SEM.

S.4. Full Experiment 1 GLMM output and comparison

Table S.1: Full output for the Experiment 1 full GLMM defined by the equation: $y \sim \beta_0 + \beta_1 I_\Delta + \beta_2 S_\Delta + \beta_3 I_\Delta S_\Delta$. Here, β_0 is the intercept term, β_1 is the weight of the pixel-wise luminance difference, I_Δ, β_2 is the weight of phase-invariant structural similarity, S_Δ, and β_3 is the weight of the interaction $I_\Delta S_\Delta$. To partially pool coefficient estimates across participants, the GLMM included participant and image combination as random effects.

Name	*Estimate*	*SE*	*tStat*	*DF*	*pValue*
Intercept	1.100	0.079	13.853	26396	<.001
S_Δ	-0.129	0.032	-4.003	26396	<.001
I_Δ	0.217	0.033	6.526	26396	<.001
$I_\Delta S_\Delta$	-0.001	0.029	-0.021	26396	.983

Table S.2: Full output for the Experiment 1 alternative GLMM defined by the equation: $y \sim \beta_0 + \beta_1 S_\Delta$. Here, β_0 is the intercept term, β_1 is the weight of the phase-invariant structural similarity, S_Δ. To partially pool coefficient estimates across participants, the GLMM included participant and image combination as random effects.

Name	*Estimate*	*SE*	*tStat*	*DF*	*pValue*
Intercept	1.139	0.079	14.342	26398	<.001
S_Δ	-0.151	0.033	-4.619	26398	<.001

Table S.3: Full output for the Experiment 1 alternative GLMM defined by the equation: $y \sim \beta_0 + \beta_1 I_\Delta + \beta_2 S_\Delta$. Here, β_0 is the intercept term, β_1 is the weight of the pixel-wise luminance difference, I_Δ, and β_2 is the weight of phase-invariant structural similarity, S_Δ. To partially pool coefficient estimates across participants, the GLMM included participant and image combination as random effects.

Name	*Estimate*	*SE*	*tStat*	*DF*	*pValue*
Intercept	1.100	0.079	13.866	26397	<.001
S_Δ	-0.129	0.032	-4.004	26397	<.001
I_Δ	0.217	0.032	6.733	26397	<.001

Table S.4: Formal model comparison. Here, we compare the three models described above, corresponding to the structural similarity-only model (S), the main effect model (M) including both structural similarity and pixel-wise luminance difference, and interaction model (I). Absolute ΔAIC, likelihood ratio statistics (LRStat), and *p* values are provided, calculated relative to the winning model (S).

Model	*DF*	*ΔAIC*	*LRStat*	*pValue*
S (Table S.2)	4	0		
M (Table S.3)	5	1.472	0.528	.467
I (Table S.1)	6	3.527	0.473	.790

Table S.5: Full output for the Experiment 1 alternative GLMM defined by the equation: $\boldsymbol{y \sim \beta_0 + \beta_1 L_\Delta + \beta_2 C_\Delta + \beta_3 L_\Delta C}$**.** Here, $\boldsymbol{\beta_0}$ is the intercept term, $\boldsymbol{\beta_1}$ is the weight of the mean luminance, $\boldsymbol{L_\Delta}$, $\boldsymbol{\beta_2}$ is the weight of the image contrast, $\boldsymbol{C_\Delta}$, and $\boldsymbol{\beta_3}$ is the weight of the interaction $\boldsymbol{L_\Delta C_\Delta}$. To partially pool coefficient estimates across participants, the GLMM included participant and image combination as random effects.

Name	*Estimate*	*SE*	*tStat*	*DF*	*pValue*
Intercept	1.025	0.079	12.980	26396	<.001
L_Δ	0.283	0.032	8.857	26396	<.001
C_Δ	0.248	0.032	7.738	26396	<.001
$L_\Delta C_\Delta$	-0.011	0.029	-0.384	26396	.701

Table S.6: Formal model comparison. Here, we compare the full interaction model described in Table S.1 (I), and the alternative model using mean luminance and image contrast described in Table S.5 (A). Absolute ΔAIC, likelihood ratio statistics (LRStat), and *p* values are provided, calculated relative to the alternative model (A).

Model	*DF*	*ΔAIC*	*LRStat*	*pValue*
A (Table S.5)	6	0		
I (Table S.1)	6	10.713	10.713	<.001

S.5. Effect of separation azimuth on Experiment 1 behavioural responses

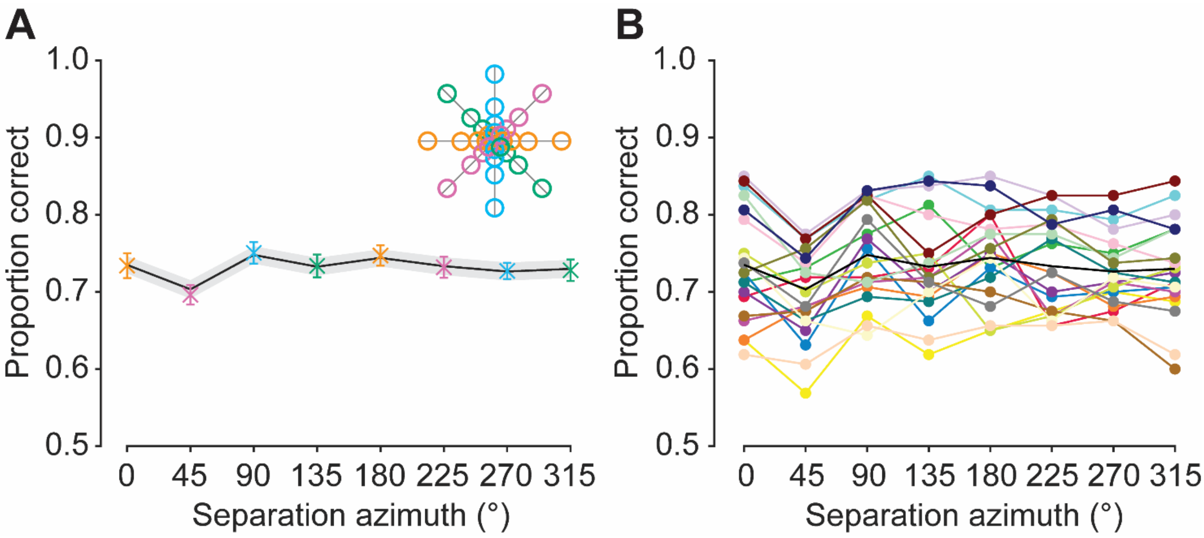

Figure S.4. Separation azimuth results for Experiment 1. A) Effect of separation azimuth on the proportion of correct responses. Data points are colour coded and are averaged across spatial locations of the same colour in the legend (inset). Solid line represents the fits of the generalised linear multilevel model outlined in **Section 3.7.2, Generalised linear multilevel modelling**. Error bars: ±1 SEM for participant responses (in some cases, standard errors are smaller than the point size). Shaded regions: ±1 SEM for trial-by-trial model predictions. **B)** Individual data showing the effect of separation azimuth on the proportion of correct responses. Solid lines are used to connect individual participants' datapoints and do not represent model fits.

S.6. Experiment 1 separation field results

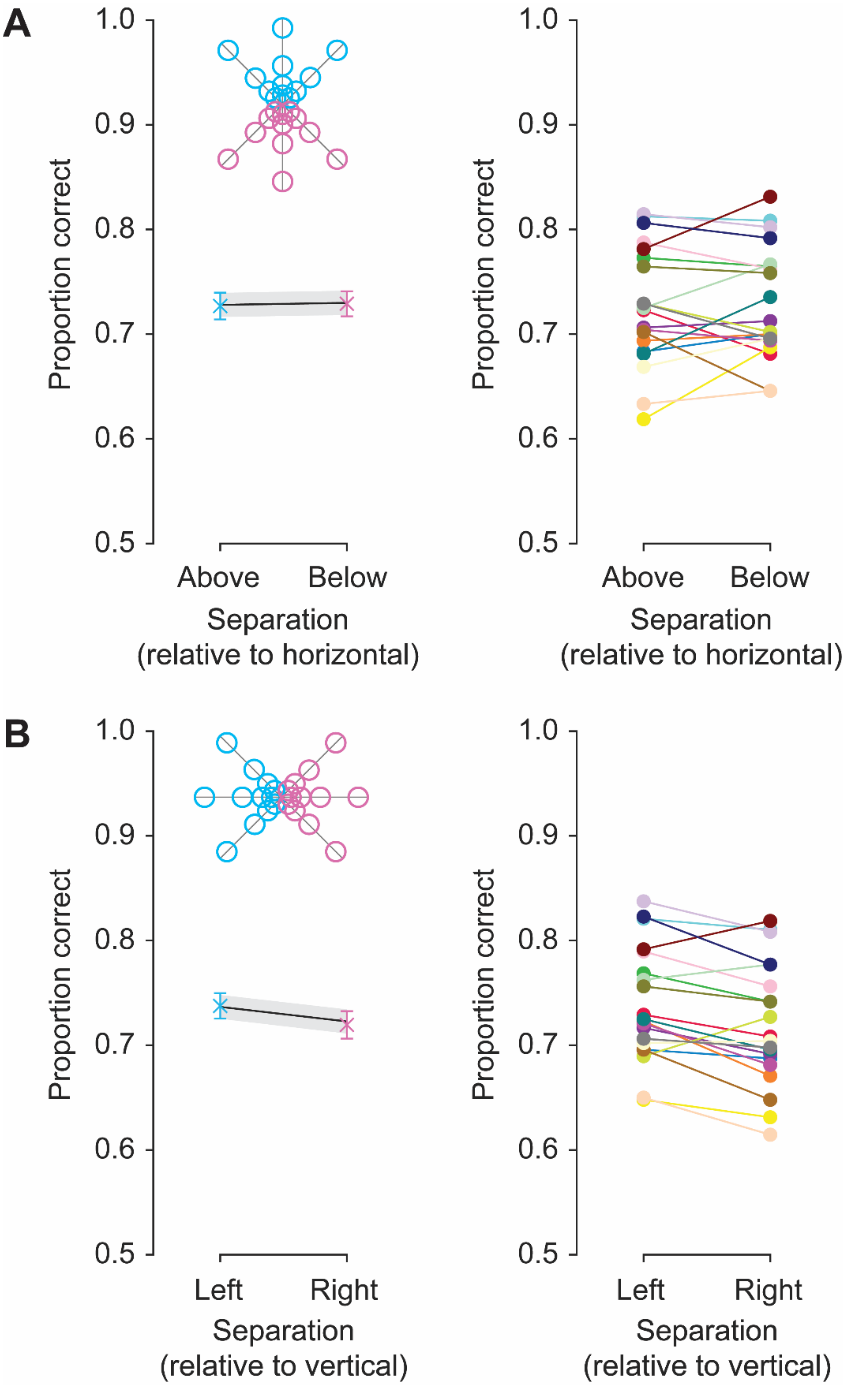

Figure S.5. Separation field results for Experiment 1. A) Effect of separation field relative to horizontal (above vs below) on the proportion of correct responses, with average (left) and individual (right) data in separate plots. Data points are colour coded and are averaged across spatial locations of the same colour in the legend above. **B)** Effect of separation field relative to vertical (left vs right) on the proportion of correct responses, with average and individual data on separate plots. Data points are colour coded and are averaged across spatial locations of the same colour in the legend above. Error bars: ±1 SEM for participant responses. Solid lines on the left plots represent the fits of the generalised linear multilevel model outlined in **Section 3.7.2, Generalised linear multilevel modelling**. Shaded regions: ±1 SEM for trial-by-trial model predictions. Solid lines on the right plots are used to connect individual participants' datapoints and do not represent model fits.

S.7. Effect of separation distance and azimuth on Experiment 2 behavioural responses

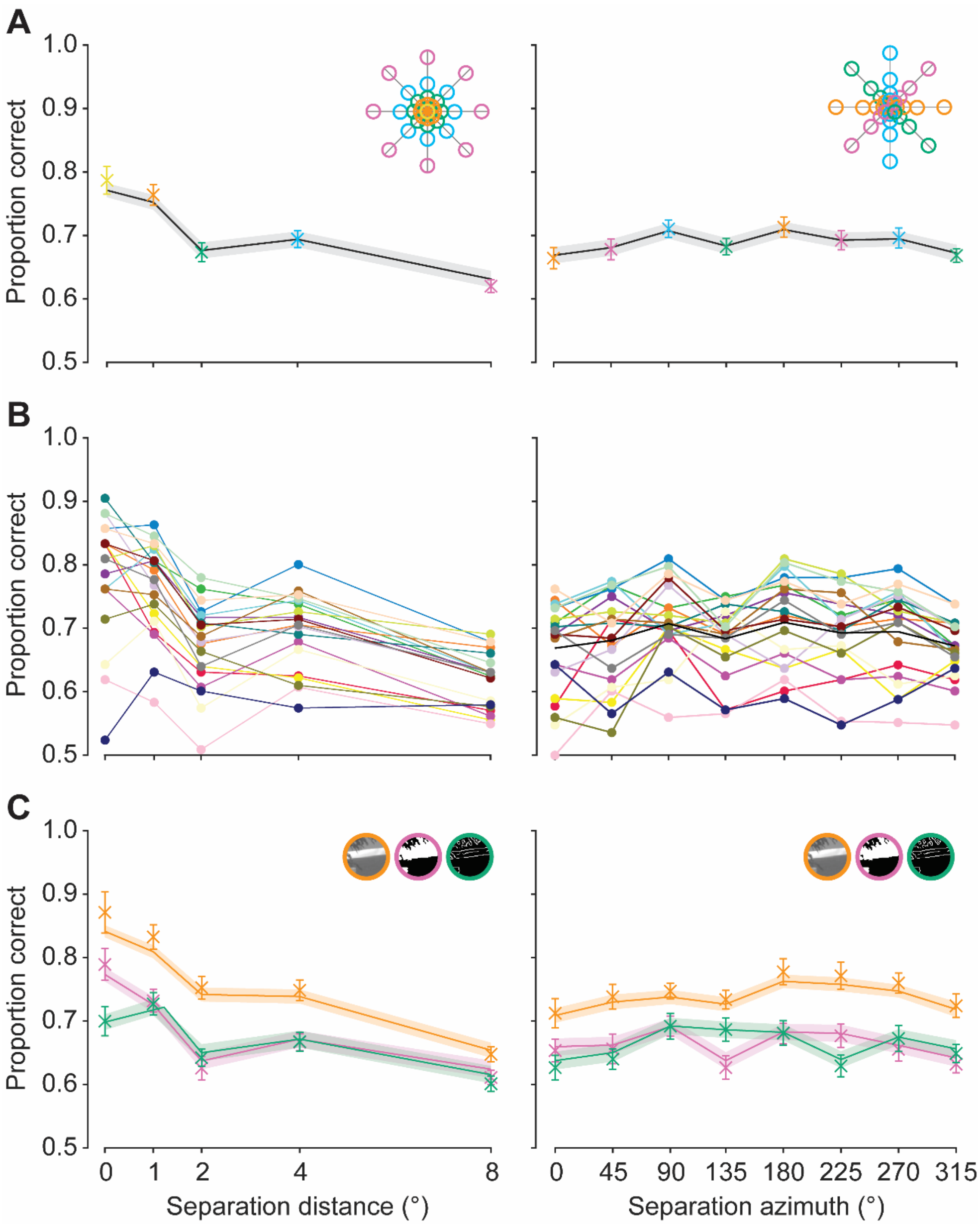

Figure S6. Separation distance (left column) and azimuth (right column) results for Experiment 2. A) Mean effect of distance (left) and azimuth (right) on the proportion of correct responses across participants. Data points are colour coded and are averaged across spatial locations of the same colour in the legend (inset, top right). Solid lines represent the fits of the generalised linear multilevel model outlined in **Section 3.7.2, Generalised linear multilevel modelling**. Shaded regions: ±1 SEM for trial-by-trial model predictions. **B)** Individual data, showing the overall effect of separation distance (left) and azimuth (right) on the proportion of correct responses. Solid lines are used to connect individual participants' datapoints and do not represent model fits. **C)** Interaction between separation distance (left)/azimuth (right) and image processing condition (separate lines, see top-right inset legend). Solid lines represent the fits of the generalised linear multilevel model outlined in **Section 3.7.2, Generalised linear multilevel modelling**. Shaded regions: ±1 SEM for trial-by-trial model predictions. Error bars: ±1 SEM for participant responses (in some cases, standard errors are smaller than the point size).

S.8. Experiment 2 separation field results

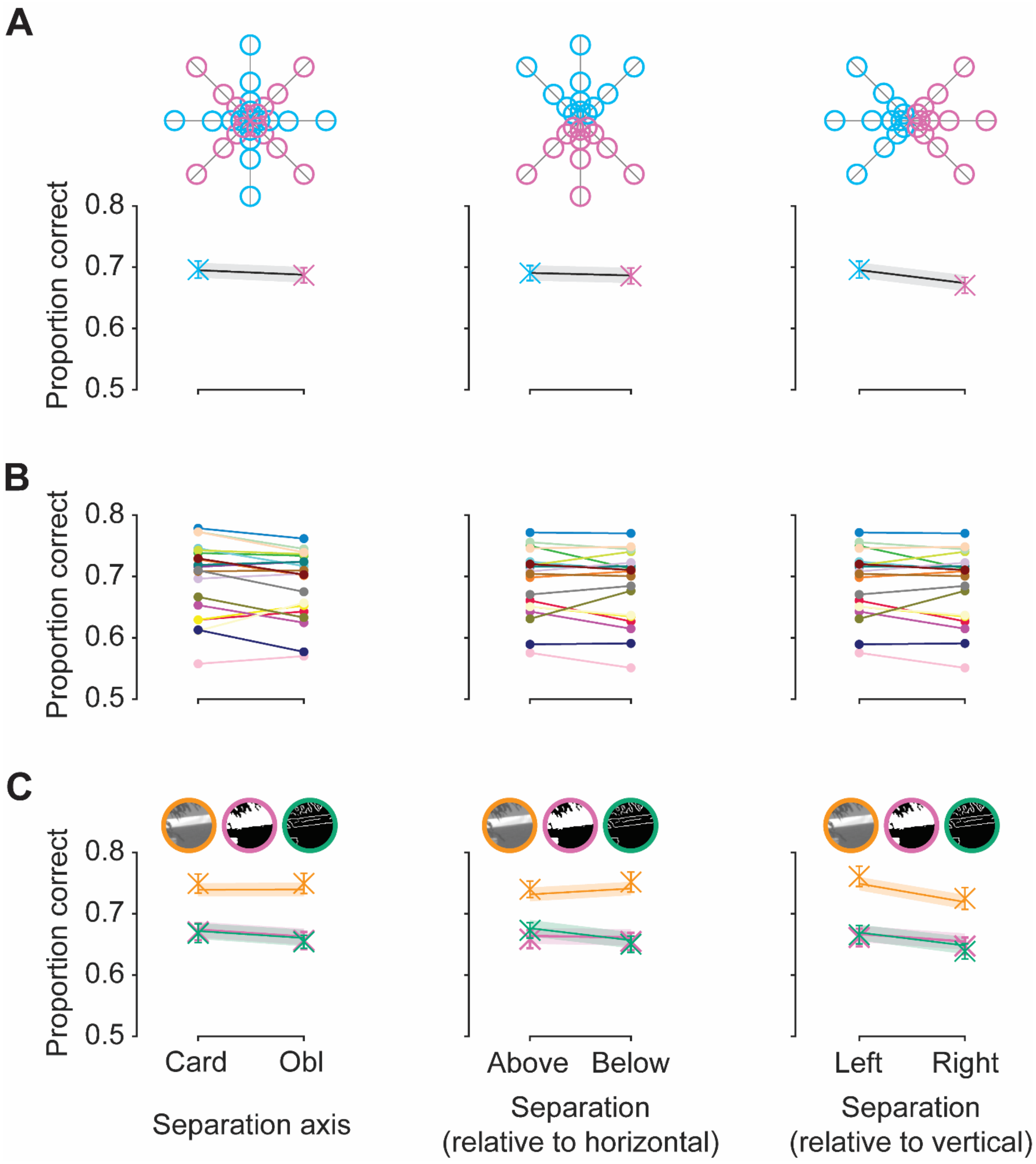

Figure S.7. Separation axis (cardinal vs oblique, left column) and field (upper vs lower, middle column; left vs right, right column) results for Experiment 2. A) Mean effect of separation axis/fields on the proportion of correct responses across participants. Data points are colour coded and are averaged across spatial locations of the same colour in the legend (inset, above). Solid lines represent the fits of the generalised linear multilevel model outlined in **Section 3.7.2, Generalised linear multilevel modelling**. Shaded regions: ±1 SEM for trial-by-trial model predictions. **B)** Individual data, showing the overall effect of v axis and fields on the proportion of correct responses. Solid lines are used to connect individual participants' datapoints and do not represent model fits. **C)** Interaction between separation axis/fields and image processing condition (separate lines, see above inset legend). Solid lines represent the fits of the generalised linear multilevel model outlined in **Section 3.7.2, Generalised linear multilevel modelling**. Shaded regions: ±1 SEM for trial-by-trial model predictions. Error bars: ±1 SEM for participant responses (in some cases, standard errors are smaller than the point size).

S.9. Experiment 2 separation distance/axis interaction

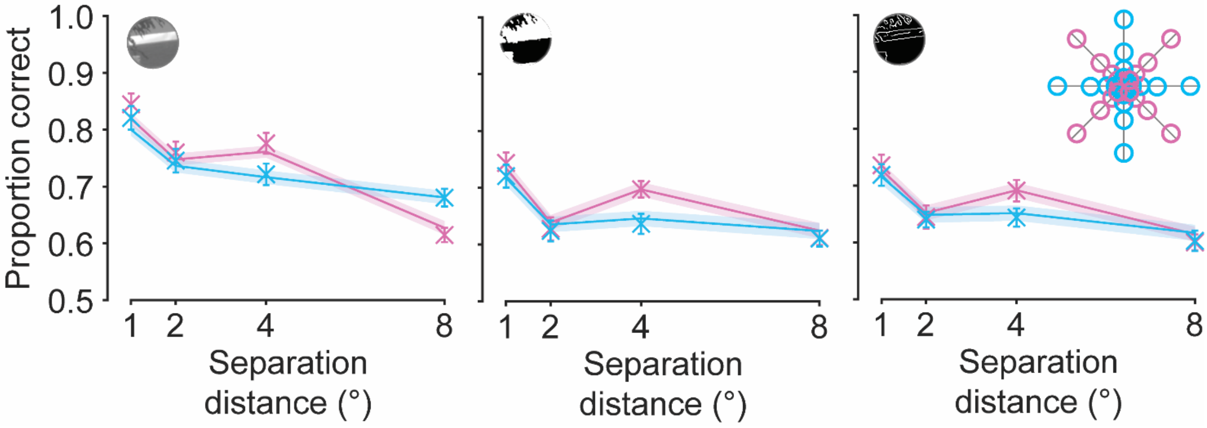

Figure S.8. Separation distance/axis interaction for Experiment 2 for different image processing conditions. Separate separation distance (x-axis) and separation axis (separate lines; see legend inset) interaction results, for each individual image processing condition (as indicated by the top left image in each plot). Solid lines represent the fits of the generalised linear multilevel model outlined in **Section 3.7.2, Generalised linear multilevel modelling**. Error bars: ±1 SEM for participant responses (in some cases, standard errors are smaller than the point size). Shaded regions: ±1 SEM for trial-by-trial model predictions.

S.10. Experiment 2 variance data

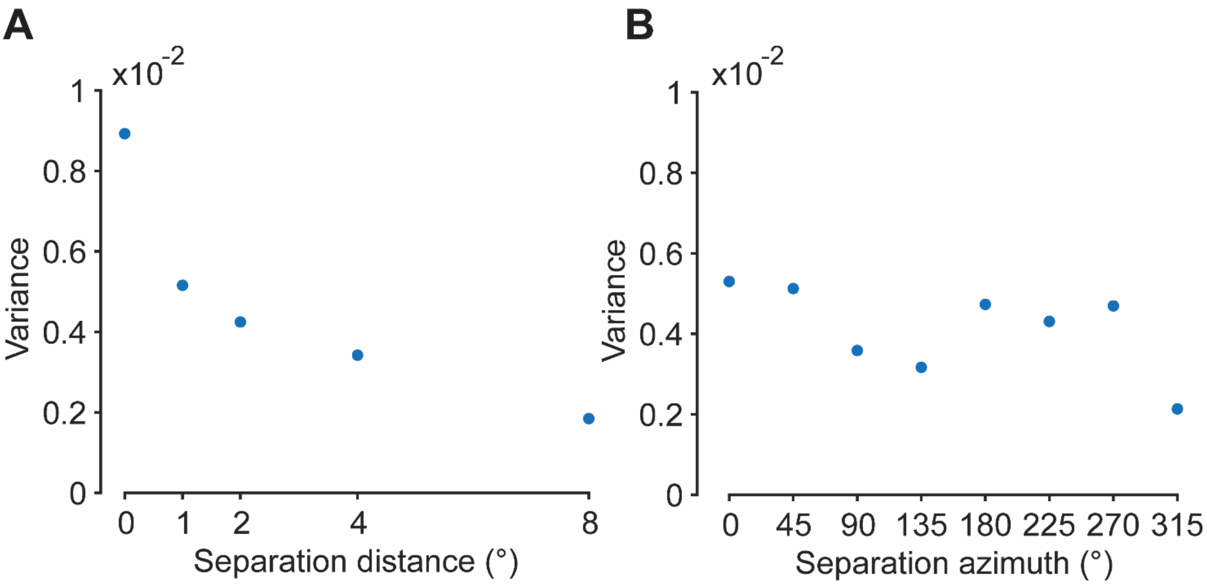

Figure S.9. Experiment 2 variance data corresponding to response accuracy data presented in Figure 6A and Figure S.6A. Plots show the effect of separation distance (**A**; x-axis) and azimuth (**B**; x-axis) on response variance (y-axis). Variance was calculated using MATLAB's var() function.

S.11. Experiment 2 individual data across image processing conditions

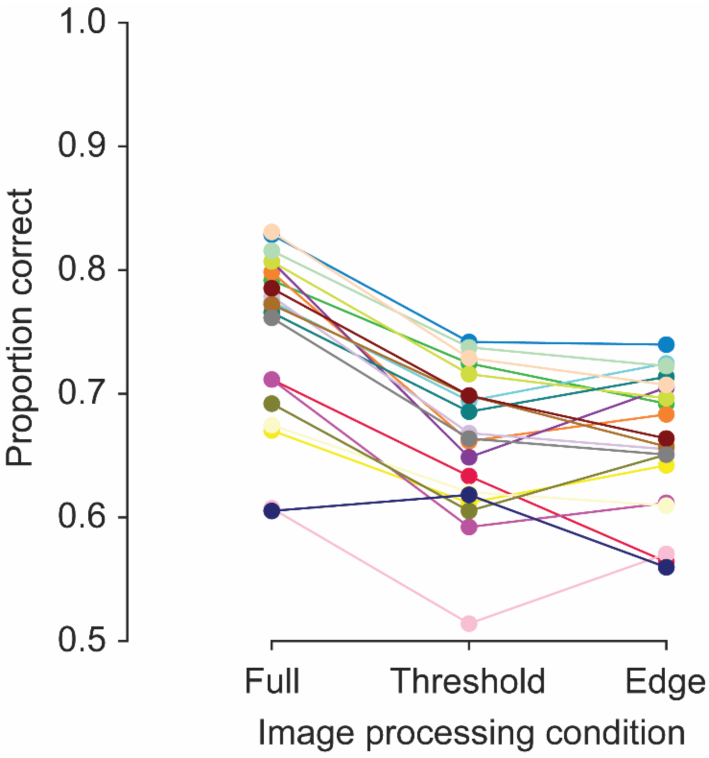

Figure S.10. Individual data corresponding to Figure 7A. The effect of image processing condition (x-axis) is plotted against the proportion of correct responses (y-axis). Solid lines are used to connect individual participants' datapoints and do not represent model fits.

S.12. Effect of separation on pixel-wise luminance error values for Experiment 2

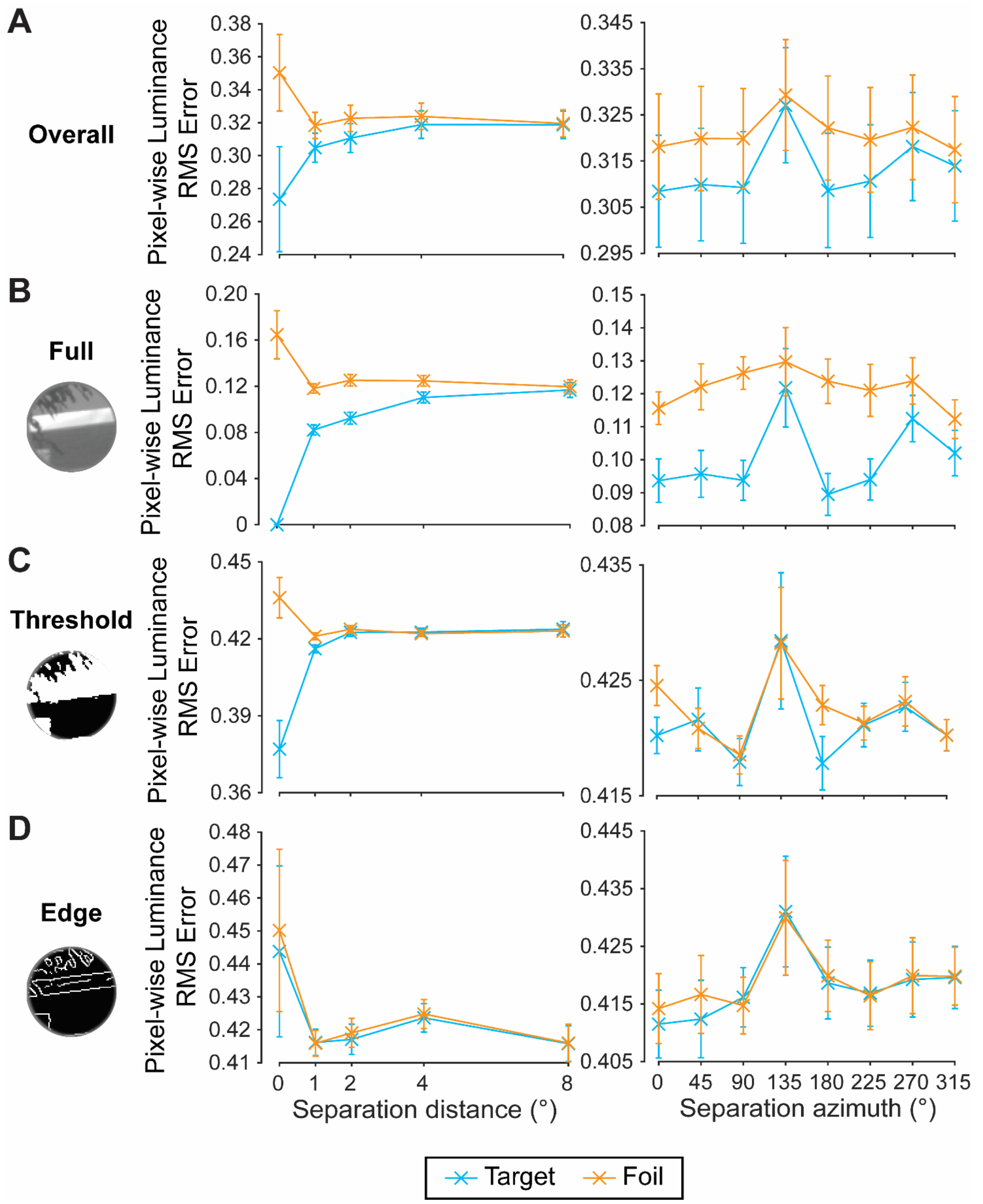

Figure S.11. Effect of separation conditions on pixel-wise luminance error values implemented for the GLMM in Experiment 2. A) Overall effect of separation distance (left) and azimuth (right) on pixel-wise luminance error values, comparing the reference patch with the target (blue) and foil (orange). **B-D)** Mean effect of separation distance and azimuth on pixel-wise luminance error values, for full, threshold, and edge image processing conditions, respectively. Each plot compares the reference patch with the target and foil patches individually. Error bars: ±1 SEM (in some cases, standard errors are smaller than the point size).

S.13. Effect of separation on phase-invariant structural similarity values for Experiment 2

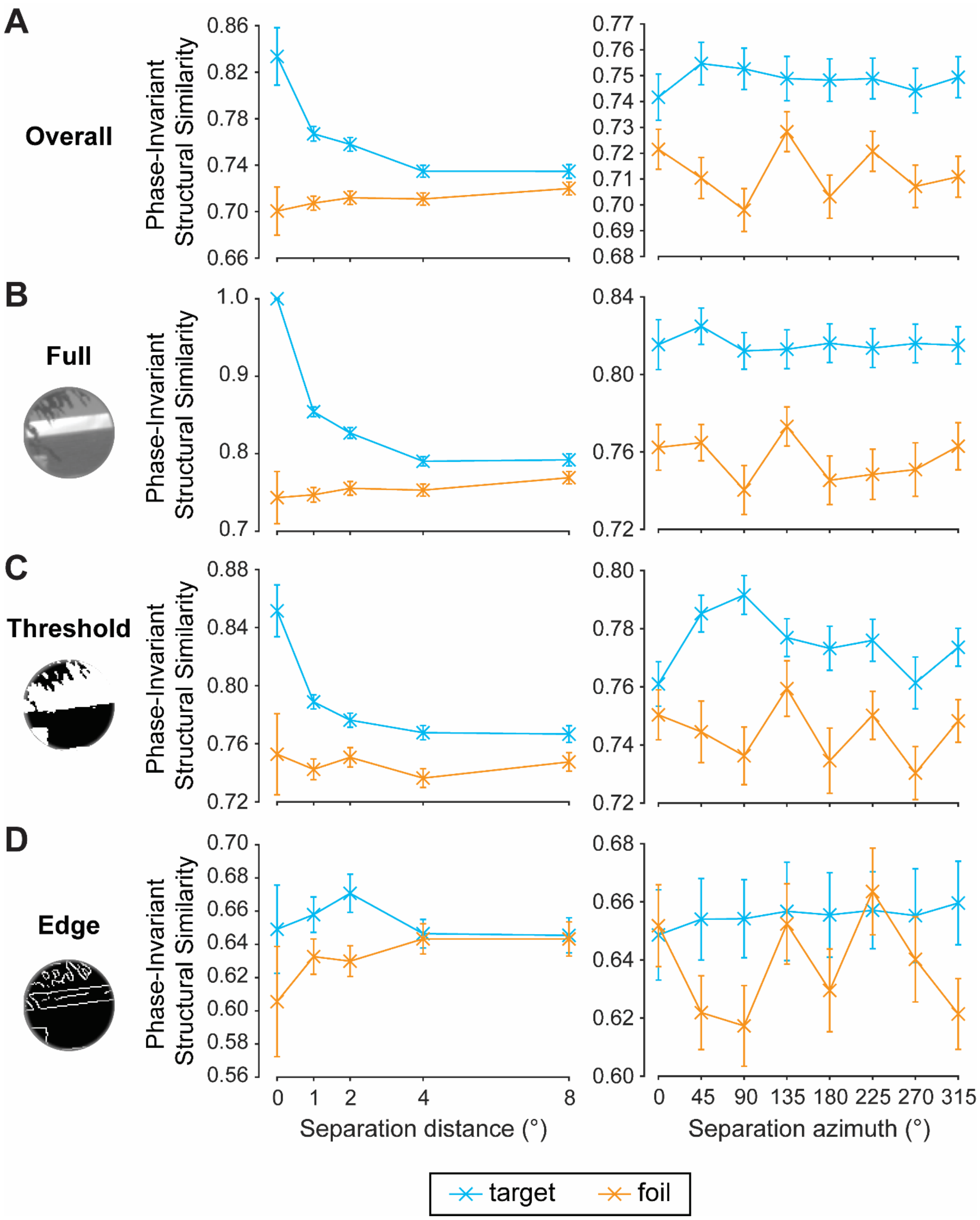

Figure S.12. Effect of separation conditions on phase-invariant structural similarity values implemented for the GLMM in Experiment 2. A) Overall effect of separation distance (left) and azimuth (right) on phase-invariant structural similarity values, comparing the reference patch with the target (blue) and foil (orange). **B-D)** Mean effect of separation distance and azimuth on phase-invariant structural similarity values, for full, threshold, and edge image processing conditions, respectively. Each plot compares the reference patch with the target and foil patches individually. Error bars: ±1 SEM (in some cases, standard errors are smaller than the point size).

S.14. Full Experiment 2 GLMM output

Table S.7: Full output for the Experiment 2 GLMM defined by the equation: $\boldsymbol{y \sim \beta_0 + \beta_1 I_\Delta + \beta_2 S_\Delta + \beta_3 I_\Delta S_\Delta}$. Here, $\boldsymbol{\beta_0}$ is the intercept term, $\boldsymbol{\beta_1}$ is the weight of the pixel-wise luminance difference, $\boldsymbol{I_\Delta}$, $\boldsymbol{\beta_2}$ is the weight of phase-invariant structural similarity, $\boldsymbol{S_\Delta}$, and $\boldsymbol{\beta_3}$ is the weight of the interaction $\boldsymbol{I_\Delta S_\Delta}$. To partially pool coefficient estimates across participants, the GLMM included participant and image combination as random effects.

Name	***Estimate***	***SE***	***tStat***	***DF***	***pValue***
Intercept	0.966	0.079	12.239	27656	<.001
S_Δ	-0.088	0.035	-2.525	27656	0.012
I_Δ	-0.031	0.035	-0.874	27656	0.382
$I_\Delta S_\Delta$	0.032	0.032	0.981	27656	0.327

Table S.8: Full output for the Experiment 2 alternative GLMM defined by the equation: $\boldsymbol{y \sim \beta_0 + \beta_1 S_\Delta}$. Here, $\boldsymbol{\beta_0}$ is the intercept term, $\boldsymbol{\beta_1}$ is the weight of the phase-invariant structural similarity, $\boldsymbol{S_\Delta}$. To partially pool coefficient estimates across participants, the GLMM included participant and image combination as random effects.

Name	***Estimate***	***SE***	***tStat***	***DF***	***pValue***
Intercept	0.960	0.079	12.181	27658	<.001
S_Δ	-0.084	0.035	-2.415	27658	0.016

Table S.9: Full output for the Experiment 2 alternative GLMM defined by the equation: $\boldsymbol{y \sim \beta_0 + \beta_1 I_\Delta + \beta_2 S_\Delta}$. Here, $\boldsymbol{\beta_0}$ is the intercept term, $\boldsymbol{\beta_1}$ is the weight of the pixel-wise luminance difference, $\boldsymbol{I_\Delta}$, $\boldsymbol{\beta_2}$ is the weight of phase-invariant structural similarity, $\boldsymbol{S_\Delta}$. To partially pool coefficient estimates across participants, the GLMM included participant and image combination as random effects.

Name	***Estimate***	***SE***	***tStat***	***DF***	***pValue***
Intercept	0.964	0.079	12.218	27657	<.001
S_Δ	-0.086	0.035	-2.478	27657	0.013
I_Δ	-0.035	0.035	-1.016	27657	0.310

Table S.10: Formal model comparison. Here, we compare the three models described above, corresponding to the structural similarity-only model (S), the main effect model (M) including both structural similarity and pixel-wise luminance difference, and interaction model (I). Absolute ΔAIC, likelihood ratio statistics (LRStat), and *p* values are provided, calculated relative to the winning model (S).

Model	*DF*	*ΔAIC*	*LRStat*	*pValue*
S (Table S.8)	4	0		
M (Table S.9)	5	1.817	0.183	.669
I (Table S.7)	6	2.275	1.725	.422

Table S.11: Full output for the Experiment 2 alternative GLMM defined by the equation: $\boldsymbol{y \sim \beta_0 + \beta_1 L_\Delta + \beta_2 C_\Delta + \beta_3 L_\Delta C}$**.** Here, $\boldsymbol{\beta_0}$ is the intercept term, $\boldsymbol{\beta_1}$ is the weight of the mean luminance, $\boldsymbol{L_\Delta}$, $\boldsymbol{\beta_2}$ is the weight of the image contrast, $\boldsymbol{C_\Delta}$, and $\boldsymbol{\beta_3}$ is the weight of the interaction $\boldsymbol{L_\Delta C_\Delta}$. To partially pool coefficient estimates across participants, the GLMM included participant and image combination as random effects.

Name	*Estimate*	*SE*	*tStat*	*DF*	*pValue*
Intercept	0.960	0.079	12.155	27656	<.001
L_Δ	0.230	0.036	6.462	27656	<.001
C_Δ	0.081	0.036	2.269	27656	.023
$L_\Delta C_\Delta$	0.076	0.030	2.538	27656	.011

Table S.12: Formal model comparison. Here, we compare the full interaction model described in Table S.7 (I), and the alternative model using mean luminance and image contrast described in Table S.11 (A). Absolute ΔAIC, likelihood ratio statistics (LRStat), and *p* values are provided, calculated relative to the full interaction model (I).

Model	*DF*	*ΔAIC*	*LRStat*	*pValue*
I (Table S.7)	6	0		
A (Table S.11)	6	1.610	1.610	<.001

S.13. GLMM output for pooled Experiment 1 and Experiment 2 data

Table S.9: Full output for the pooled data GLMM defined by the equation: $\boldsymbol{y \sim \beta_0 + \beta_1 I_\Delta + \beta_2 S_\Delta + \beta_3 I_\Delta S_\Delta}$**.** Here, $\boldsymbol{\beta_0}$ is the intercept term, $\boldsymbol{\beta_1}$ is the weight of the pixel-wise luminance difference, $\boldsymbol{I_\Delta}$, $\boldsymbol{\beta_2}$ is the weight of phase-invariant structural similarity, $\boldsymbol{S_\Delta}$, and $\boldsymbol{\beta_3}$ is the weight of the interaction $\boldsymbol{I_\Delta S_\Delta}$. To partially pool coefficient estimates across participants, the GLMM included participant and image combination as random effects.

Name	***Estimate***	***SE***	***tStat***	***DF***	***pValue***
Intercept	1.036	0.057	18.202	54056	<.001
S_Δ	-0.109	0.023	-4.797	54056	<.001
I_Δ	0.094	0.023	4.014	54056	<.001
$I_\Delta S_\Delta$	0.022	0.021	1.020	54056	0.308
